# 2D Materials for Gas Sensing Applications: A Review on Graphene Oxide, MoS_2_, WS_2_ and Phosphorene

**DOI:** 10.3390/s18113638

**Published:** 2018-10-26

**Authors:** Maurizio Donarelli, Luca Ottaviano

**Affiliations:** 1 Sensor Laboratory, Department of Information Engineering, University of Brescia, Via Branze 38, 25136 Brescia, Italy; 2 Department of Physical and Chemical Sciences, University of L’Aquila, Via Vetoio 10, 67100 L’Aquila, Italy; luca.ottaviano@aquila.infn.it; 3 CNR-SPIN, UOS L’Aquila, Via Vetoio 10, 67100 L’Aquila, Italy

**Keywords:** graphene oxide, MoS_2_, WS_2_, phosphorene, gas sensors

## Abstract

After the synthesis of graphene, in the first year of this century, a wide research field on two-dimensional materials opens. 2D materials are characterized by an intrinsic high surface to volume ratio, due to their heights of few atoms, and, differently from graphene, which is a semimetal with zero or near zero bandgap, they usually have a semiconductive nature. These two characteristics make them promising candidate for a new generation of gas sensing devices. Graphene oxide, being an intermediate product of graphene fabrication, has been the first graphene-like material studied and used to detect target gases, followed by MoS_2_, in the first years of 2010s. Along with MoS_2_, which is now experiencing a new birth, after its use as a lubricant, other sulfides and selenides (like WS_2_, WSe_2_, MoSe_2_, etc.) have been used for the fabrication of nanoelectronic devices and for gas sensing applications. All these materials show a bandgap, tunable with the number of layers. On the other hand, 2D materials constituted by one atomic species have been synthetized, like phosphorene (one layer of black phosphorous), germanene (one atom thick layer of germanium) and silicone (one atom thick layer of silicon). In this paper, a comprehensive review of 2D materials-based gas sensor is reported, mainly focused on the recent developments of graphene oxide, exfoliated MoS_2_ and WS_2_ and phosphorene, for gas detection applications. We will report on their use as sensitive materials for conductometric, capacitive and optical gas sensors, the state of the art and future perspectives.

## 1. Introduction

Gas sensing is becoming more and more important in our society, due to the need to quickly identify toxic gases and organic vapours, for environmental and human security, for the emission control, in industry sector and medical diagnosis. Conducting polymers [1,2,3], carbon nanotubes [4,5] and metal oxides, in many forms (thick or thin films, nanorods, nanowires, etc.) [6,7], have been widely used to detect target gases. Metal oxides in particular, have shown the best characteristics, in terms of responses and sensibility, among the other materials. Furthermore, their low cost and ease of fabrication, make them widely used in gas sensing devices. However, the resistive metal oxide-based gas sensors usually work at high temperatures (higher than 100 °C), leading to a high power consumption and the high operating temperatures deals to drifts in gas sensing responses, due to the growth of metal oxide grains. Another drawback of the metal oxide based gas sensors is their lack of selectivity [8,9,10]. Conducting polymers based gas sensors are able to work at room temperature (RT), however their sensing properties are affected by relative humidity and the storage in air can lead to degradation [11,12,13]. Therefore, the gas sensing community’s efforts are devoted to the research of new materials, which are able to detect gases at RT, in standard environmental conditions and have high selectivity and sensibility.

**Figure 1:**
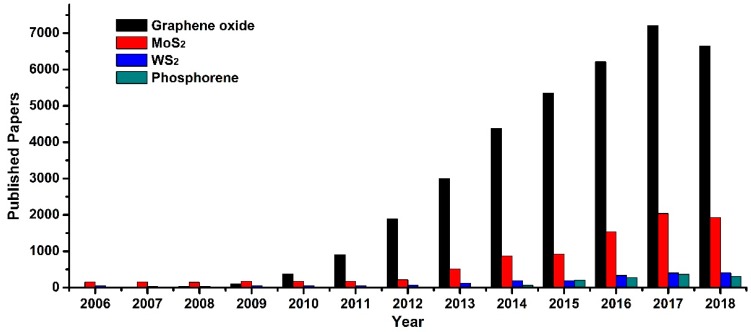
Number of published papers vs. year of publication for “graphene oxide”, “MoS_2_”, “WS_2_” and “phosphorene” or “exfoliated black phosphorus”. (Source: Scopus, 28 September 2018).

An atom-thick film of sp^2^ carbon atoms, called graphene, was conceptualized in 1947 [14] and synthesized in the first year of this century [15]. Its outstanding morphological characteristics and its fascinating electronic properties (zero band-gap, high RT carrier mobility of about 200,000 cm^2^V^−1^s^−1^) [16,17,18] immediately attract the attention of the researchers worldwide. Small concentrations of target gases adsorbed on the graphene surface can cause a sensible change of its resistance, which suggested its use as a very sensitive material for gas detection applications [19]. The easy exfoliation routes to obtain graphene leads to its wide use for fabrication of gas sensing devices. After the discovery of graphene, many other 2D materials (like exfoliated molybdenum disulfide, MoS_2_, or exfoliated tungsten disulfide, WS_2_, or exfoliated black phosphorus, phosphorene) have been synthesized and investigated. Many of them have been synthesized by a top-down approach: their 3D counterparts are formed by many layers, weakly bonded by van der Waals forces, allowing an easy mechanical or wet chemical exfoliation. The 2D materials field is now one of the main topic in the material science, physics of matter, chemical engineering and sensing. In Figure 1, the number of published papers with the word “graphene oxide”, “MoS_2_”, WS_2_” and “phosphorene” or “exfoliated black phosphorus” is reported. In the last decade, a tremendous increase of interest can be noticed. 

Due to their morphological properties, 2D materials appear soon as promising candidates for gas sensors, having an intrinsically high surface-to-volume ratio. Furthermore, 2D materials different from graphene also show semiconductive properties, with direct or indirect bandgap, tunable with the number of layers [20,21]. Graphene oxide, that is, oxidized graphene, has been the first graphene-like 2D material investigated and the research on its gas sensing properties and performances had a tremendous increase in the last years. Scopus (at September 2018) records about 880 papers on “graphene oxide gas sensors”, with an exponential increase from 2007. Graphene oxide synthesis and investigation has been followed by exfoliated MoS_2_ in the first 2010’s. As for graphene and graphene oxide, its morphological characteristics have been exploited for the fabrication of gas sensors. MoS_2_ opens the doors to the discovery and isolation of novel 2D nanostructures of sulphides and selenides, like WS_2_, WSe_2_, MoSe_2_ and so forth [22,23,24]. On the other hand, 2D materials constituted of just one atomic species have been successfully synthesized [25], like one layer thick black phosphorus (phosphorene) [21,26] or silicon (silicene) [27,28] or germanium (germanene) [29].

In this review, we will report a comprehensive resume of the recent developments in 2D materials-based gas sensors. Excellent reviews on 2D materials for gas sensors have been recently published [30,31,32,33]. Differently from these reviews, we mainly focus and discuss only graphene oxide, MoS_2_, WS_2_ and phosphorene, going deeply into their use as sensitive materials for gas detection and summarize the latest results. Furthermore, we will describe their use not only in chemiresistor and FET devices (which are the most widespread types of gas sensing devices) but also in impedance, optical and quartz crystal micro-balances gas sensors. In the second section, different kinds of gas sensing devices are reported. The following sections are devoted to the above mentioned 2D materials, each of them being the focus of one section. In the last section, we try to conclude and discuss the future perspectives on the use of 2D materials for gas sensing applications.

## 2. Gas Sensing Devices

When exposed to different target gases, the sensing materials can change their electronic, electrical and optical characteristics. These changes constitute the sensing signal. In particular, the conductivity of the device increases when the n- (p-) type sensing layer is exposed to reducing (oxidizing) gases, like CO, ethanol, hydrogen, etc. (NO_2_, ozone, SO_2_, etc.). These effects on the conductivity of the sensing material exposed to different gases can be due to two concurrent mechanism. In particular in metal oxide-based sensors, at OT higher than 100 °C, oxygen ions (O_2_^−^, O^−^ and O^2−^) adsorb on the surface of the sensitive layer. The target gases will interact with these oxygen ions: for example, in the case of CO, CO interaction with the oxygen ions results in its oxidation, in the form of CO_2_, and one electron will be released on the surface of the metal oxide, increasing its conductivity, in the case of n-type material, or decreasing its conductivity, in the case of p-type material. In the case of an acceptor gas, like NO_2_, the gas molecules will accept the charge, leading to a decrease of the conductivity for n-type materials and an increase of the conductivity for the p-type materials [34,35]. The other mechanism does not involve the adsorbed oxygen ions: the target gas molecules adsorbed on the surface of the sensitive layer and a charge transfer reaction occurs, with different directions and quantity of charge, due to the acceptor or donor behaviour of the target gas and of the sensitive material. Leenaerts et al. analysed the charge transfer mechanism between graphene and various gases. They found that H_2_O and NO_2_ act as acceptor and NH_3_, CO and NO act as donor when adsorbed on the graphene sheet [36].

In this section, a description of the sensing performance parameters and a (not exhaustive) resume of the main types of gas sensing devices are reported.

### 2.1. Sensing Performance Parameters

The sensors performances can be described by several parameters: sensor response, limit of detection, operating temperature, response and recovery times, selectivity to a certain gas and stability. The sensor response is defined as the relative change of the sensing signal when the target gas is injected into the test chamber. In the simplest case of resistive device, as described before, the sensor response is the electrical resistance relative change. This parameter is strictly related to the limit of detection of the sensors, which is the lowest target gas concentration that the sensor is capable to detect. Usually, in gas sensing applications, the limit of detection is the minimum concentration the sensor can detect, with a signal to noise ratio equal to 3:1. For example, the U.S. EPA has set the NO_2_ exposition limit for one hour at 100 ppb [37], therefore, the NO_2_ limit of detection for gas sensors should be under this value. The operating temperature of the gas sensors is another key parameter for their use and commercialization. Metal oxide-based sensors usually work at high temperature (higher than 100 °C). At these temperature, the oxygen molecules are adsorbed on the surface (or, at temperatures higher than 200 °C, chemisorbed on the surface) and interact with target gas molecules, leading to high responses. Furthermore, high operating temperatures allow fast response and recovery times. However, working at high operating temperatures increases the power consumption of the devices and, in some cases, can lead to a change in the sensing behaviours of the metal oxide [38,39]. Therefore, the gas sensing research, in the last years, is devoted to the fabrication of sensors working at room temperature, reducing the power consumption and without the need of providing a heater to the sensors. Latest results on the use of 2D materials for RT gas sensing are promising, exploiting their morphological and electronic properties. Response time is usually defined as the time required for the sensor signal to change from its value before the gas injection to the 90% of the final value during the gas injection. Vice versa, the recovery time is the time required for the sensor signal to recover the 90% of its value before the gas injection [40]. These values can be in the range of one second- tens of minutes. High response and recovery times values can represent a hurdle for the use of sensors in everyday life. The selectivity is the ability of a sensor to respond to a certain gas, in the presence of other gases. In normal conditions, the sensors are exposed to a mix of gases, therefore, the selectivity of a sensor can be estimated by exposing it to different target gases and recording the different responses. The stability of the response is another key parameter of the gas sensors. The response of the device should not change over time (months, years). This issue is not always dealt with in the scientific papers; however, it is very important for the engineering of the devices [41]. The stability of the response can be affected by chemical change in the sensing layer, for example oxidation when exposed to air (we will show that this is a very important issue for phosphorene). All these parameters have to be taken into account for the choice of the best gas sensor in a given situation or environment. The analysis of these parameters can distinguish between a “good” and a “bad” gas sensor.

### 2.2. Chemiresistors

Chemiresistors are very likely the most used kind of gas sensing devices, due to their simplicity of operation and fabrication, low cost and power consumption, ability to reuse. Their operating principle is based on the fact that adsorbed gas molecules on the sensitive layer can change its electrical resistance (as explained before). In order to track these changes, the sensitive layer is deposited between two or more interdigitated metal electrodes on an insulating substrate (alumina, silicon dioxide, quartz, etc.). This kind of sensors are widely used also for metal oxide layers, which need high operating temperatures, therefore many chemiresistors are equipped with a heater (usually a metal coil exploiting the Joule’s effect) or the sensing layers are deposited on a micro-hot plate to heat the device up to the optimal operating temperature.

**Figure 2:**
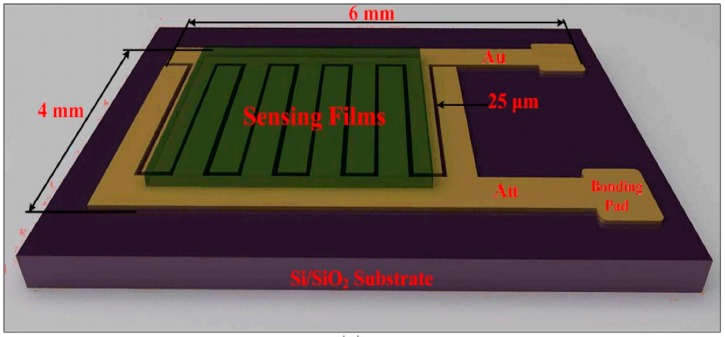
Schematic illustration of a chemiresistor (adapted from ref. [42], Copyright 2013, with permission from Elsevier, Amsterdam, The Netherlands).

In Figure 2 is reported a chemiresistor fabricated by using drop casted graphene-polyaniline (G-PANI) composite (green area) as sensing layer [42]. The G-PANI is deposited on interdigitated gold electrodes, deposited on a SiO_2_ insulating substrate. The electrodes are 25 µm spaced. The reported device has been used to detect NH_3_ in a 1–6400 ppm range. The response, as usual for the chemiresistors, has been calculated following the formula:

$$\mathrm{Response}=\frac{R_{G}-R_{\mathrm{air}}}{R_{\mathrm{air}}}\times 100\%$$

where R_G_ and R_air_ are, respectively, the resistance of the device when exposed to NH_3_ and in clean air.

In this case, the sensing tests have been performed at 25 °C, in a N_2_ dry environment. The response of the sensor is linear with the concentration of NH_3_. The response and recovery times are, respectively, 50 s and 23 s.

### 2.3. Field Effect Transistors (FETs)

**Figure 3:**
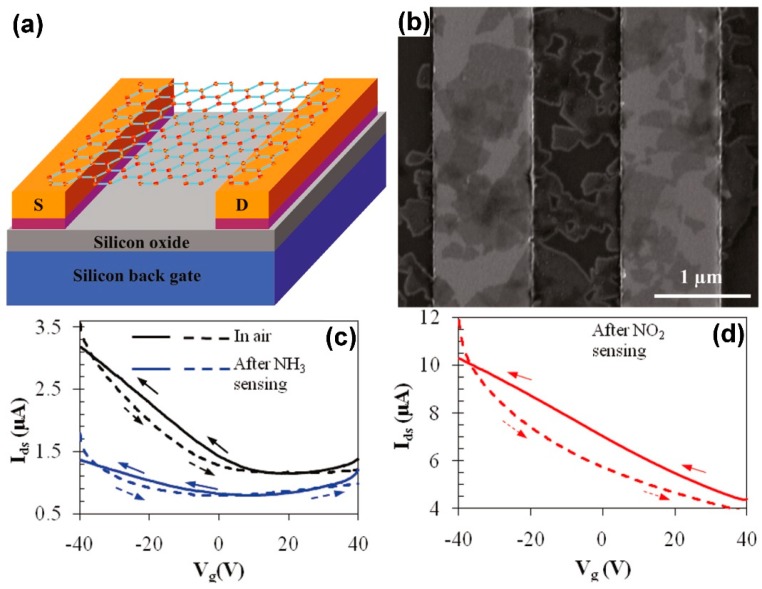
Panel (**a**): schematic illustration of a FET sensor based on reduced graphene oxide; Panel (**b**): SEM image of the device, the brightest regions are the metal electrodes; Panel (**c**): I_ds_ vs. V_g_ curves before (black curve) and after (blue curve) exposure to NH_3_; Panel (**d**): I_ds_ vs. V_g_ curve after exposure to NO_2_ (adapted with permission from [43]. Copyright 2011, American Chemical Society, Washington, DC, USA).

Another type of gas sensors widely used is the field-effect transistor (FET). As the chemiresistors, FETs are low cost, low power consuming, easy to fabricate and to miniaturize devices. In the typical FET scheme, the sensing semiconductive layer constitutes the channel, between two electrodes, (source and drain). The conductance of the channel can be modulated by a voltage applied to the gate electrode through a thin dielectric layer. The target gas can be detected by measuring the conductance changes of the semiconductive channel (i.e., the changes of the drain-source current), due to the electronic structure changes induced by the adsorbed gas molecules on the surface of the semiconductor. In Figure 3, a FET gas sensing device is reported [43].

A layer of chemically reduced graphene oxide has been deposited on two metal electrodes (source, S, and drain, D, Figure 3a) and backgated through a thin SiO_2_ layer. A drain-source voltage of 0.1 V is applied and the gate voltage (V_g_) is varied between −40 V and +40 V. The drain-source current (I_ds_) versus V_g_ is reported in Figure 3c,d. When exposed to air, the device shows a typical p-type conductivity (Figure 3c, black curve), while, after exposure to NH_3_, the I_ds_ versus V_g_ curve is V-shaped (Figure 3c, blue curve), indicating an ambipolar conductance and a n-type doping effect of the NH_3_ molecules, acting as reducing agents. After NO_2_ sensing (Figure 3d) the I_ds_ slope is higher than in air, suggesting a strong p-type doping induced by the NO_2_ oxidizing molecules adsorbed on the surface of reduced graphene oxide. Furthermore, the authors show that the drain-source current decreases when the device is exposed to NH_3_ and it increases when exposed to NO_2_. Therefore, being the reduced graphene oxide a p-type semiconductor, the sensing mechanism in this FET device working at RT is mainly based on the charge transfer between gas molecules and reduced graphene oxide.

### 2.4. Impedance Sensors

Impedance sensors are less diffused that the previously described gas sensing devices. The design of the impedance sensors is similar to the ones reported before: the sensing layer is deposited between metal electrodes. A sinusoidal voltage is applied between the electrodes, with frequency ranging from sub-Hz to MHz (different from microwave gas sensors, working in the ten of MHz-GHz range [44]) and the measured electrochemical impedance spectrum is the sensing signal (while in the case of microwave gas sensors, the rough response is the reflected wave). In particular, the impedance phase and absolute value can be monitored. Furthermore, a device equivalent circuit can be modelled and the values of its constituting elements can be monitored during the gas sensing tests, giving more “sensing signal” (for example, if the equivalent circuit is a RC circuit, the values of the resistance and of the capacitance in the whole frequency range can be evaluated before and after the target gas injection). This kind of device is promising to detect sub-ppm concentrations of hydrocarbons, NO_x_, CO and humidity. The study of the impedance spectrum of these sensors can improve the selectivity, adding other parameters to the data analysis, useful to discriminate between different gas species.

**Figure 4:**
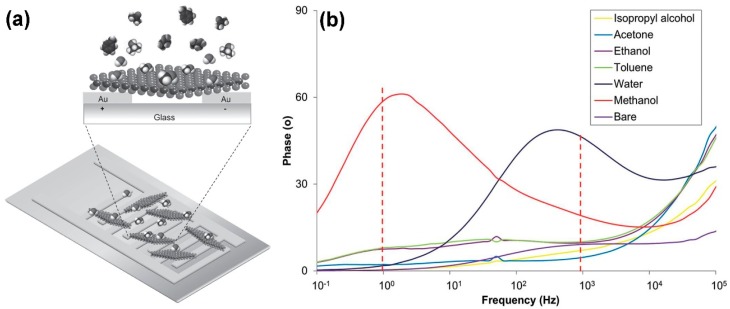
Panel (**a**): schematic of the impedance sensor with a 1T-WS_2_ sensing layer; Panel (**b**): selectivity studies of 1T-WS_2_ sensor, impedance phase spectra (adapted with permission from [45]. Copyright 2015, John Wiley and Sons, Hoboken, NJ, USA).

An impedance sensor based on an exfoliated 1T-WS_2_ layer (which is metallic, instead of semiconductive) is reported in Figure 4a [45]. The authors demonstrate that the impedance phase spectra of 1T-WS_2_ present specific resonant frequencies for methanol and water vapours (Figure 4b), respectively at 1 Hz and 1 kHz, therefore these characteristics can be used to improve the selectivity of the device.

### 2.5. Optical Gas Sensors

The sensing signal is usually related to a change of the electrical properties and characteristics of the sensing material. Also, the change of optical properties (absorbance, fluorescence, reflectivity, etc.) of the sensing layer can be exploited to detect target gases. In this review, we will call “optical gas sensors” those devices whose signal is given by a change of some optical characteristics.

**Figure 5:**
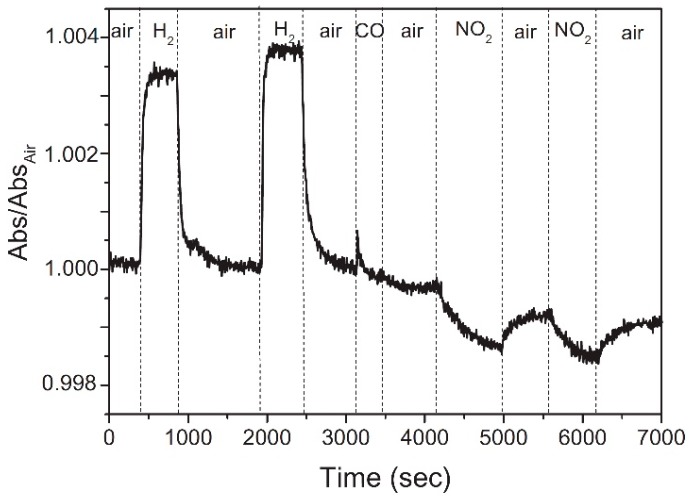
Absorbance change of rGO/Au NPs sample exposed to 10,000 ppm H_2_, 10,000 ppm CO and 1 ppm NO_2_. The incident wavelength is 528 nm (adapted from [46], Copyright 2013, with permission from Elsevier, Amsterdam, The Netherlands).

For example, in Figure 5, the absorbance relative changes of reduced graphene oxide (rGO) deposited on a gold nanoparticles (NPs) monolayer are reported [46]. The absorbance of the rGO/Au NPs increases when exposed to H_2_ (reducing gas) and decreases when exposed to NO_2_ (oxidizing gas).

Surface plasmon resonance (SPR) sensors are sensing devices which exploit the optical properties of the material. An incident light beam excites the surface plasmon of the material and a detector collects the reflected or diffracted light. A change in the refractive index of the sensing layer changes the characteristics of the incident light for SPR excitation. This kind of sensors are used in particular for biological molecules and in the last years graphene, graphene oxide and MoS_2_ have been used to fabricate SPR sensors [47,48,49,50]. The photoluminescence spectrum, of some of its features, can be used as sensing signal, analysing its shift or intensity changes during target gas injection.

### 2.6. Quartz Crystal Microbalance (QCM) Gas Sensors

Applying voltage to a quartz crystal leads to its oscillation a certain frequency (the resonant frequency). The change in mass on the quartz surface causes a change of this frequency. A quartz crystal microbalance (QCM) is constituted by a thin quartz disk, cut to a specific orientation and equipped with gold electrodes. Sauerbrey [51] found that adsorbed mass on the surface of the quartz crystal leads to a decrease of the resonant frequency, according to the equation:

$$∆f=-\frac{2f_{0}^{2}}{A\sqrt{\rho_{q}\mu_{q}}}∆m$$

where Δf is the variation of the resonant frequency f_0_, A is the piezoelectrically active area, Δm is the mass change, ρ_q_ is the density of quartz and µ_q_ is the shear modulus of cut quartz. In a QCM gas sensor, different Δf are associated to the detection of analytes with different molecular weight and concentrations. QCM coated with a specific sensitive layer can adsorb specific target gas molecules and the concentration of that gas can be estimated. The sensitivity of a QCM gas sensor is related to its thickness: thin QCMs lead to high resonant frequencies and high sensitivities [52]. 

For example, graphene films have been grown by CVD on a Cu foil and then transferred onto a QCM [53]. The so-fabricated device can detect buthanol, isopropanol, acetone and ethanol at RT. The target gas molecules adsorb on the defect sites of the graphene sheet, leading to a change of the resonant frequency of the QCM, which can be monitored. The graphene-coated QCM sensor shows excellent reproducibility and low response and recovery times (less than 100 s).

## 3. Graphene Oxide and Reduced Graphene Oxide Sensors

**Figure 6:**
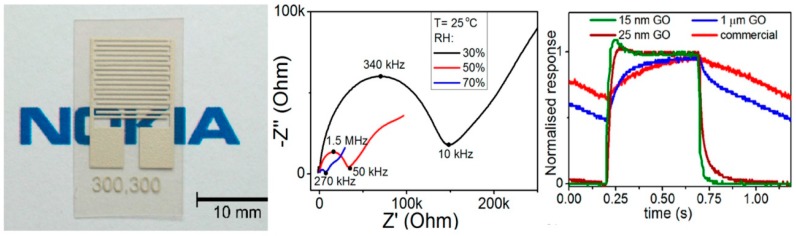
(**Left panel**) photograph of the sprayed GO on Ag electrodes. Only the Ag electrodes are visible, due to the transparency of the deposited ultrathin GO film; (**Central panel**) Nyquist plots of the GO flakes recorded at different RH values; (**Right panel**) response of three GO sensors with different heights to wet air, compared with an ultrafast commercial sensor (adapted with permission from ref. [69]. Copyright 2013, American Chemical Society, Washington, DC, USA).

Graphene has attracted great attention for gas sensing applications due to its morphological characteristics, especially its high surface to volume ratio. However, its zero or quasi-zero bandgap can represent a hurdle to use it as sensitive layer in devices. Therefore, many authors have proposed to functionalize and/or decorate graphene [54,55,56,57,58,59,60,61]. The easiest way is to use the graphene oxide (GO). GO flakes can be easily produced, in high quality and quantity, from graphite oxide. Graphite oxide can be obtained by treating graphite with strong oxidizers. The layered structure of graphite is conserved, however the interlayers spacing is higher than in graphite. When graphite oxide is dispersed in basic solutions, the bulk material is exfoliated, leading to single layer GO flakes, which are highly soluble in water. The main route to fabricate graphitic oxide and after that GO flakes, is the Hummers’ method [62], which has been improved, in order to obtain less defective and wider GO flakes and to increase the yield of production [63,64,65,66,67,68]. GO flakes have been widely used for detection of relative humidity (RH) changes. Spray deposited GO flakes have been used to fabricate an impedance sensor (Figure 6, left panel), showing very fast humidity response (up to 30 ms) and working at room temperature or near room temperature [69]. The Nyquist plots reported in Figure 6, central panel, clearly show that the GO flakes impedance is dependent on the RH values. 15 µm thick GO flakes layer has been demonstrated to be the best choice for the fastest humidity sensors, exploiting the intrinsically 2D nature of GO flakes (Figure 6, right panel).

In a recent work, the dielectric constant (both real and imaginary parts) of GO flakes have been used as the sensing signal [70]. The real and imaginary parts of the GO dielectric constant increase with increasing RH, in the GHz regime. The GO flakes have been printed on a graphene RFID antenna: the change of the dielectric properties of the GO induces a change in the resonance and in the impedance of the antenna, while the conductance increase of the GO is negligible respect to the conductance of the graphene. In this way, a prototype of battery-free humidity sensor is fabricated. Another example about the use of impedance spectroscopy to detect RH changes is reported in ref. [71]. Here the sensing signal is constituted by the equivalent capacitance values obtained fitting the recorded Nyquist plots. The GO-based sensor works at 25 °C and 1 kHz, with an ultrahigh response (up to 37,800%) and very high stability, although the response and recovery times are of the order of tens of seconds. Decreasing the GO dimensions can favour the decrease response and recovery times of the capacitive sensors to RH changes. GO flakes dispersed in water have been used to fabricate a CMOS compatible device, able to detect RH changes [72]. The capacitance variations constitute the sensing signal. The authors fabricated a prototype device, which has a dedicate integrated circuit at the PCB level.

Li et al. [73] demonstrated a sub-second response and recovery times of a GO quantum dots-based sensor and they proposed it as a device to monitor the human breathing. Also in this case, the authors proposed to check the impedance to detect RH changes. Few works deal with GO foam for humidity sensing at RT [74,75]. The authors monitored the impedance, dielectric loss and permittivity changes to detect the RH changes.

A more exotic way to detect RH changes using GO flakes is reported by Yao et al. [76]. In this work, the authors deposited few drops of a water/GO flakes solution on a micromachined silicon bridge, with a fully piezoresistive Wheatstone bridge embedded in it. When exposed to humidity, the GO flakes swell, leading to a bending of the membrane, which can be measured by the resistance change of the piezoresistive components constituting the Wheatstone bridge. The recorded output voltage of the system is the sensing signal.

**Figure 7:**
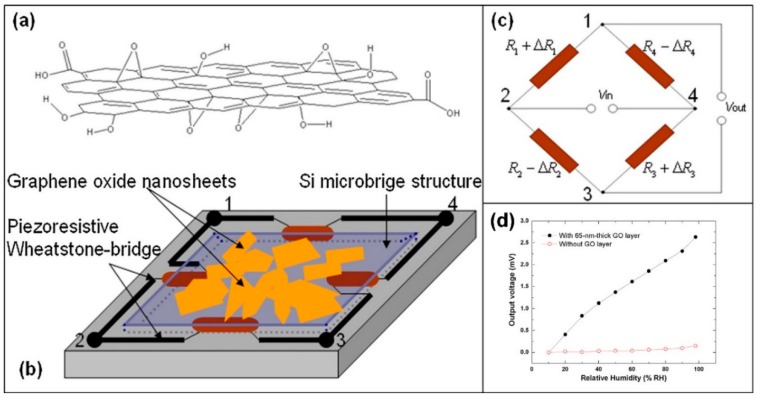
Panel (**a**): chemical structure of GO flakes; Panel (**b**): schematic image of the GO flakes deposited on the Si membrane and the embedded Wheatstone bridge; Panel (**c**): piezoresistive Wheatstone-bridge circuit; Panel (**d**): response curve to humidity of the 65 nm thick GO layer deposited on the Si microbridge (black curve) and of the bare Si microbridge (red curve) (adapted from [76], Copyright 2012, with permission from Elsevier, Amsterdam, The Netherlands).

In Figure 7, a schematic image of the GO flakes deposited on the Si membrane (panel (b)) and the embedded Wheatstone bridge is reported (panel (c)). The output voltage is a monotonic curve: it increases when the RH increases (Figure 7, panel (d)).

Exploiting the GO flakes swelling when exposed to humid atmosphere, they have been deposited on quartz crystal micro-balances (QCM) for humidity sensing [77,78]. It was demonstrated that the GO-coated QCM have higher frequency stability and higher Q-factor than polyethylene glycol-covered QCM. Furthermore, the frequency shifts are monotonically related to changes of RH, with little hysteresis and good reproducibility.

Other strategies to detect RH changes include the exploitation of GO flakes optical properties. GO flakes have been deposited with inkjet spray technique on a tilted fibre Bragg grating (TFBG) with a diameter of 20 µm [79]. Increasing the RH, a shift of the resonance peak around 1535 nm (in the third optical communications window) to lower wavelengths is observed. The sensitivity is −0.01 nm/%RH and the linearity is 0.996.

**Figure 8:**
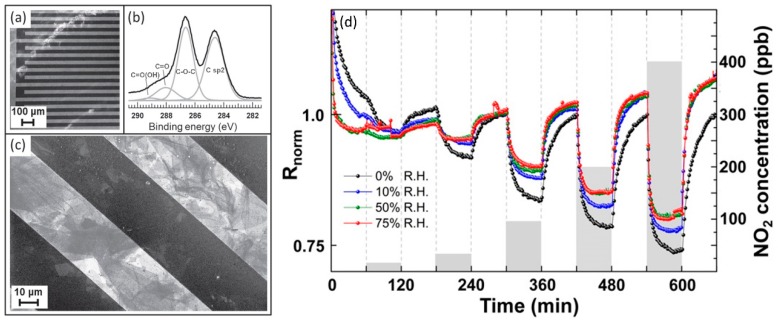
Panel (**a**): SEM image of the device. The lighter stripes are the Pt interdigitated electrodes on Si_3_N_4_ substrate; Panel (**b**): XPS C 1s core level spectrum of the deposited GO flakes. The grey lines are the fitting curves, labelled with their own relative C chemical bond (adapted with permission from [80]. Copyright 2013 American Chemical Society, Washington, DC, USA); Panel (**c**): SEM image of the device at higher magnification than (**a**); Panel (**d**): normalized resistance of a GO-based conductometric gas sensor exposed to various NO_2_ concentrations (ranging from 20 to 400 ppb) at different RH (adapted with permission from [81], © IOP Publishing, Bristol, United Kingdom. Reproduced with all permission. All rights reserved.).

Conductometric devices, in which tens of µm sized GO flakes bridge the metal interdigitated electrodes, can detect the change from dry to wet atmosphere, at 150 °C operating temperature. A SEM image of the GO based conductometric device is reported in Figure 8, panel (a). The flakes, analysed by XPS, are well oxidized (Figure 8, panel (b)) and, due to their big size, they can “bridge” Pt interdigitated electrodes (Figure 8, panel (c)). These devices increase their resistance passing from dry to humid air, as a typical p-type semiconductor [80,81]. However, the relative resistance change is not dependent on the RH values (when different from 0%). Far from be a flaw, this characteristic is very useful for the detection of other target gases, for example NO_2_: the NO_2_ sensing signal does not change with different RH values (different from 0%), therefore these devices can be used also in standard conditions [81]. Figure 8 reports the normalized resistance of the device when exposed to various NO_2_ concentrations at different RH.

The GO ability to detect NO_2_ gas is strictly related to the oxygen functional groups on its surface. A comparison between the responses (in terms of resistance change) to NO_2_ at RT of GO-, reduced GO- and graphene-based sensors has been conducted [82]. It reveals that GO shows higher responses to NO_2_ (while graphene sensor is not sensitive) and, different from reduced GO sensor, it can recover the baseline after the sensing cycles. While the GO sensing behaviour is usually reported as p-type, some authors report a n-type sensing behaviour of GO flakes. In particular, the GO n-type behaviour has been observed for GO flakes deposited on pre-patterned substrates by dielectrophoresis method [83]. The dielectrophoresis assembled GO flakes decrease their resistance when exposed to H_2_, a reducing gas, which is a fingerprint of n-type conductivity. The dielectrophoresis parameters are crucial to obtain high response value and the devices can detect H_2_ in dry air environment in a range between 100–1000 ppm.

Also, the optical properties of GO have been used to detect molecules. Here, we focus on the gas sensing properties of GO, therefore this review will not concern on optical biosensing of GO and other 2D materials about which refer to more specific reviews [84,85] and references therein. Knowing that VOCs can affect the reflectance of GO flakes, a polymer optical fibre tip has been coated with GO flakes and inserted in a sensing chamber, at RT and in humid conditions [86]. As for the conductometric gas sensor reported in ref. [81], the optical sensing of the VOCs is not affected by the humidity and the GO flakes decrease their reflectance when exposed to hydrazine, methanol, ethanol, acetone, THF, nitromethane and diethylamine.

**Figure 9:**
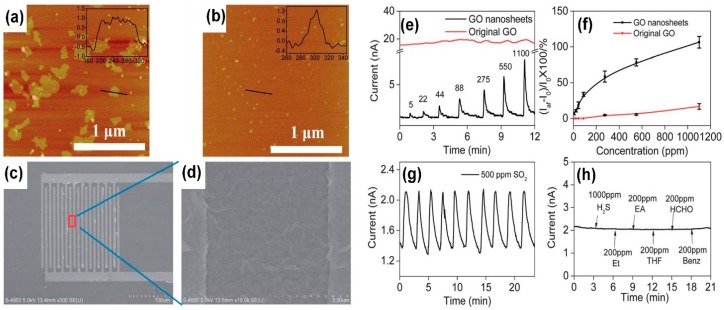
Panel (**a**): AFM image of the pristine GO flakes. The height profile of a flake is reported in the inset; Panel (**b**): AFM image of the tailored GO flakes. The height profile of a flake is reported in the inset; Panel (**c**): SEM image of the interdigitated electrodes on the FET device; Panel (**d**): SEM image of the GO flakes bridging the electrodes; Panel (**e**): response to different concentrations of SO_2_ of pristine GO (red curve) and tailored GO flakes (black curve); Panel (**f**): current vs. SO_2_ concentration graph of the pristine GO (red curve) and tailored GO flakes (black curve); Panel (**g**): ten cycles of the tailored GO flakes for response to 500 ppm of SO_2_; Panel (**h**): real time response of the tailored GO flakes to other gases. (Adapted and reproduced with permission of RSC Pub., Cambridge, United Kingdom, from [87]; permission conveyed through Copyright Clearence Center, Inc.).

In order to increase the sensing performances, in particular to increase the selectivity of the devices to a specific gas, many authors proposed to modify the GO surfaces, functionalizing or micromachining them. Tailoring the edges of GO flakes can lead to an improvement of the sensing ability of the GO-based device to certain gas. GO flakes, fabricated with a modified Hummers’ method and then exposed to periodic acid, decrease their lateral size and the edges are endowed with quinoid groups [87]. The tailored GO flakes can detect SO_2_ at RT, differently from pristine GO (Figure 9e) and, furthermore, the response is reproducible (Figure 9g) and they are selective to SO_2_ (Figure 9h).

**Figure 10:**
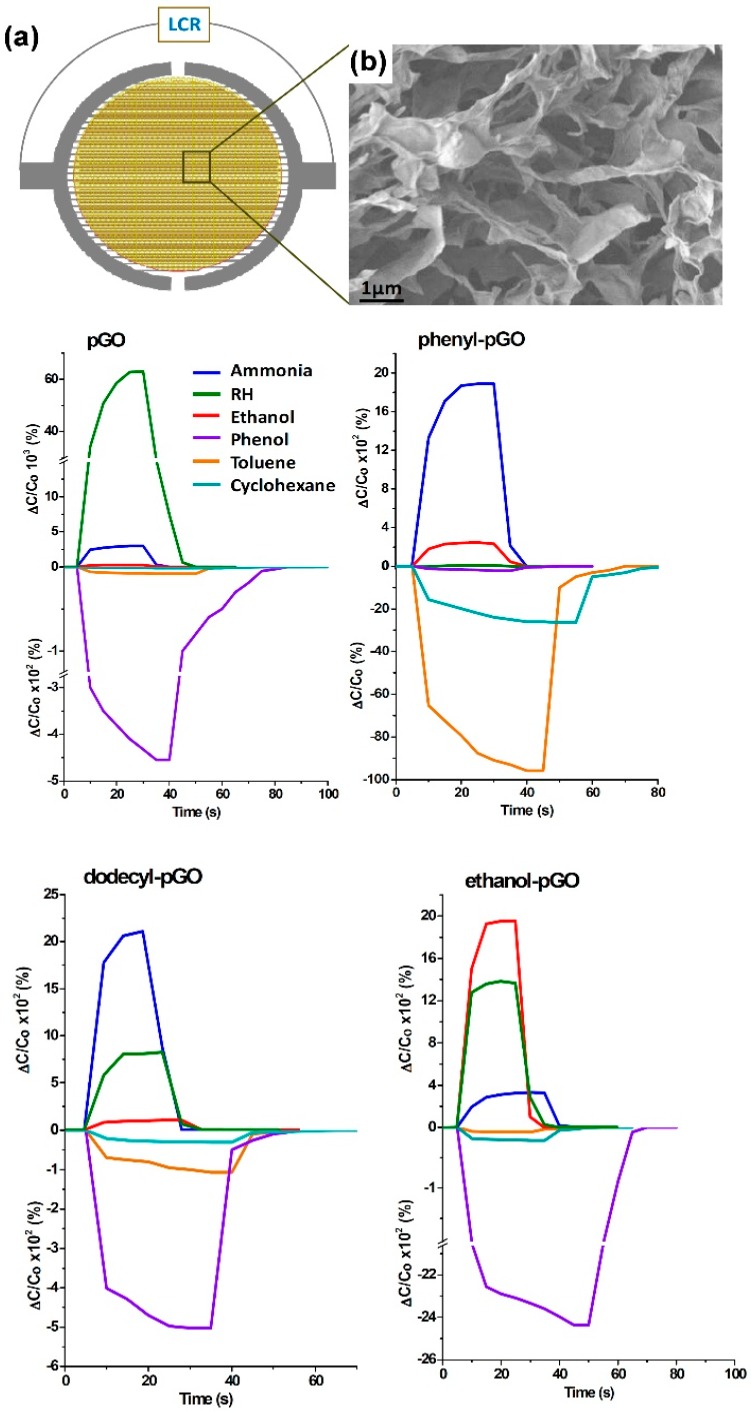
Panel (**a**): sketch of the device showing the electrodes and the dielectric porous GO (pGO) between them; Panel (**b**): SEM image of the pGO network. The graphs report the responses of the not-functionalized (pGO) and phenyl-, dodecyl-, ethanol-functionalized GO sensors to different gas vapours, indicated according to colour code (concentration 180 ppm) and 75% RH. (Adapted and reproduced with permission of RSC Pub., Cambridge, United Kingdom, from [95]; permission conveyed through Copyright Clearence Center, Inc.).

Functionalization of the exposed surfaces has been observed to be very useful for selective detection of gases and to obtain higher responses, in particular for metal oxide (MOX)-based gas sensors [88,89,90,91,92,93]. Functionalized GO flakes have been synthesized and used to obtain higher gas sensing responses and more selective gas sensors. A chemiresistive gas sensor, based on chemically fluorinated GO flakes, has been fabricated [94]. The fluorine adatoms enhance the gas sensor’s ability to detect NH_3_ at RT, reaching a detection limit of about 6 ppb. Porous GO have been used to fabricate capacitive gas sensors [95]. The porous GO sensor exhibits the ability to detect RH changes and NH_3_. The authors have functionalized the porous GO scaffold, fabricating phenyl-GO, dodecyl-GO and ethanol-GO, in order to obtain selective gas sensing. Each functionalized and not-functionalized sensor has been exposed to 180 ppm of different vapours. The recorded results are reported in Figure 10.

Functionalized GO flakes with amine-silica NPs have been deposited to QCM sensors and they show sensitivity to formaldehyde [96].

GO flakes show high resistivity, therefore, to partially restore the graphene conducibility, usually they are reduced, obtaining reduced graphene oxide (rGO) flakes. Many ways to reduce graphene oxide have been reported in the last years. A widely used method implies the use of hydrazine [67,97,98,99,100]. Other authors proposed the use of NaBH_4_ at 125 °C for three hours to obtain a partial reduction of GO flakes [64]. GO flakes can be reduced also by exposure to hydrogen plasma for few seconds [101] or by thermal annealing [102,103,104]. Optical approaches have been used to deoxygenate the GO surface and to selective pattern GO flakes, with laser, UV lamp, Xenon lamp flashes, EUV laser and synchrotron radiation [105,106,107,108,109,110,111].

rGO flakes have been largely used for gas sensing, taking advantage from the presence of residual hydroxyl groups on the flakes surfaces and the partially restored graphene conducibility. Hydrazine reduced GO flakes have been demonstrated to be good molecular sensors. Robinson et al. [112] have showed that the reduction degree, controlled by the exposure time to hydrazine hydrate vapours of the GO flakes, can tune the sensing properties of the rGO-based device. They showed that rGO sensor can detect pulses of chemical warfare agents at ppb level, at RT. RT operating devices are of big technological interest, in order to decrease the power consumption of the devices and to safely use them in potentially explosive atmosphere. Other researchers have tried to chemically reduce GO flakes with agents different from the widely used hydrazine. For example, GO flakes, self-assembled on gold contacts, have been reduced with pyrrole vapour and hydrazine vapour. The NH_3_ sensing tests at RT show that the pyrrole reduced GO flakes have higher response than hydrazine reduced ones to NH_3_ concentrations ranging from 5 ppb to 100 ppm [113]. Also, NaBH_4_ has been used to reduce GO flakes, in order to fabricate sensors for the selective detection of NH_3_ at RT [114]. The device has been exposed to various NH_3_ concentrations, in dry N_2_ atmosphere, showing quite high selectivity towards ammonia. The sensing mechanism is based on the capability of the functional groups and defects on the flakes surfaces to be active sites for the target gases adsorption. The high number of functional groups and defects increases the sensing response; however, it affects the recovery times, which decreases with the increase of the sp^2^ bonds, that is, with a higher reduction of GO flakes. Therefore, it is important to find the best compromise between the sensing responses and the recovery times, tuning the GO reduction time in NaBH_4_. Thermal annealing in inert atmosphere can reduce the GO flakes. Thermally reduced GO flakes in Ar have been reported to be able to detect toxic gases, like NO_2_ and NH_3_, at RT [115]. Also in this case, the sensing behaviour and the response and recovery times are dependent on the reduction degree, namely, the higher the annealing temperature, the faster the response of the rGO sensor. All these devices show a p-type sensing behaviour, like the pristine GO flakes. rGO flakes have been demonstrated to be good sensitive layers for the detection of NO_2_. Holey rGO flakes, fabricated with hydrothermal treatment at 150 °C and used as sensitive layer for a chemiresistive device, show a detection limit of 60 ppb NO_2_ [116]. The presence of nanostructured holes on the surface of rGO flakes, increases the surface area and, combined with residual functional groups, provides many adsorbing sites for NO_2_ molecules, leading to high responses.

**Figure 11:**
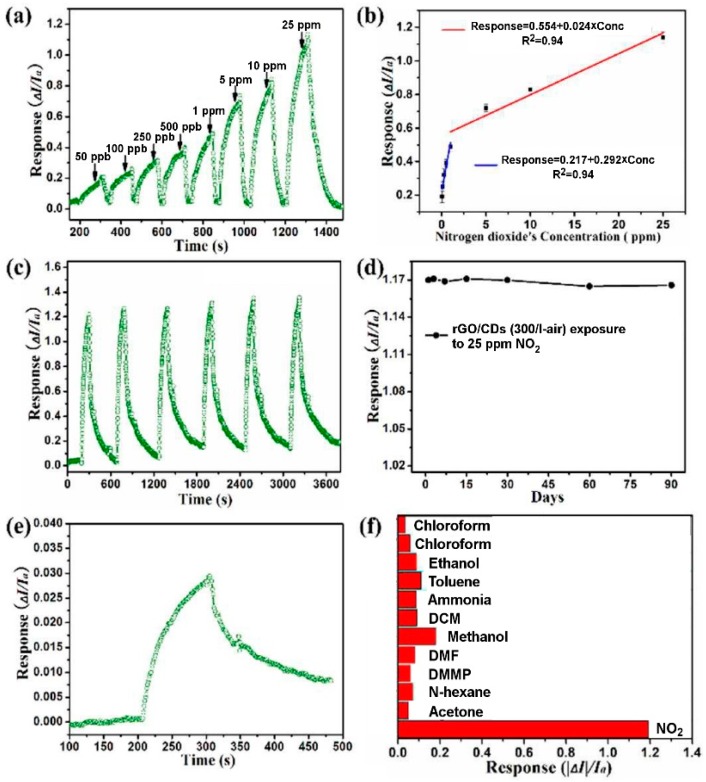
Panel (**a**): response of the rGO-CDs sensor to NO_2_ concentrations ranging from 50 ppb to 25 ppm; Panel (**b**): calibration curve of the rGO-CDs sensor vs. NO_2_ concentrations; Panel (**c**): reproducibility tests of the rGO-CDs sensor; Panel (**d**): stability of the sensor response over 90 days; Panel (**e**): response curve upon exposure to 10 ppb of NO_2_; Panel (**f**): selectivity of the rGO-CDs sensor: all the bars but the first and the last, are the response of the sensor to 2% of the saturated vapour pressure (SVP) of the labelled gas; the first to 1% SVP chloroform and the last to 25 ppm of NO_2_. (Adapted and reproduced with permission of RSC Pub., Cambridge, United Kingdom, from [121]; permission conveyed through Copyright Clearence Center, Inc.).

In order to be wearable, sensors on flexible substrates (plastic, organic, etc.) have been fabricated. GO flakes have been inkjet on flexible plastic with previously fabricated electrodes and then reduced with ascorbic acid, obtaining a “green” sensor [117]. The NO_2_ molecules strongly chemisorb on the rGO flakes, leading to a very low detection limit of about 400 ppt. The NO_2_ interaction with the rGO flakes leads to a decrease of the device resistance, that is, a p-type behaviour. Another stretchable rGO-based conductometric gas sensor has been fabricated and tested in standard conditions, differently from the previous one, which has been tested only in chambers with controlled environment [118]. The hydrazine-reduced rGO have been deposited on a polyurethane nanofibres. The sensor shows a low detection limit (50 ppb), demonstrating, again, the strong chemisorption of NO_2_ molecules on the rGO flakes surfaces. However, the sensor response is highly related to the strain and the surrounding environments, therefore it needs improvements to be used in every-day life. The implementation of optically reduced (by Xe lamp flashes) GO flakes, deposited on a network of Ag nanowires embedded in a polymide substrate, was one of the first flexible and transparent NO_2_ conductometric sensor, with 5 ppm detection limit [119]. The strong adsorption of NO_2_ molecules on the rGO flakes surface is very useful to obtain a high response and low detection limit of the fabricated conductometric devices. However, the main problem arising from this characteristic is the very slow desorption of NO_2_ molecules from the surface, leading to high recovery times, which constitute a big problem for effective gas sensors. The researchers proposed to expose the rGO flakes to UV light, to help the desorption of NO_2_ molecules from the sensing layer. Furthermore, the exposition to UV and visible light has been demonstrated to be useful for the selective detection of NO_2_ and, for example, SO_2_ in a conductometric gas sensor based on a g-C_3_N_4_/rGO heterostructure [120]. Heterostructure interfaces, due to their capability to promote charge transfer, have been proposed for the fabrication of conductometric devices able to detect NO_2_ at RT. Hu et al. show that a heterostructure composed of rGO flakes and carbon nanodots, deposited on a interdigitated patterned substrate, can detect up to 10 ppb of NO_2_ with a response (calculated as $\frac{I_{a}-I_{g}}{I_{a}}$, where I_a_ and I_g_ are, respectively, the current in dry air and the current during NO_2_ exposure) of 74.3% for 5 ppm of NO_2_. They also demonstrate that this sensor has a high selectivity to NO_2_ towards other vapours [121] (Figure 11).

Other kind of devices, different from the usual conductometric ones, have been developed to detect NO_2_. Here we cite, as an example of them, an optical gas sensor based on rGO flakes deposited on an etched fibre Bragg Grating, which can detect NO_2_ concentrations ranging from 0.5 to (at least) 3 ppm, in dry air and at RT [122]. The sensing signal is constituted by the Bragg shift (>10 pm for 0.5 ppm of NO_2_), due to the change of the rGO refractive index caused by the charge transfer between rGO flakes and NO_2_ molecules adsorbed on them.

The increasing interest in detection of hazardous gases at RT deals the researchers to test rGO as sensing material for the detection of CO_2_ and NO. The possibility to detect CO_2_ with a resistive device based on highly reduced GO flakes, has been demonstrated [123]. The charge transfer between CO_2_ and graphene (coming from the reduction of GO flakes) allows to detect CO_2_ in air at standard humidity conditions and, furthermore, the low adsorption strength of CO_2_ on flakes surface results in easy desorption of gas molecules without light assistance. Another resistive device, with electrodes made of CVD grown graphene and Pd-decorated rGO flakes as sensing layer, has been used to detect NO [124]. The tests are far from standard conditions (they have been performed in N_2_, at RT with Ar flow for the recovery), however, the authors demonstrated the possibility of the rGO flakes, adequately decorated and coupled with graphene electrodes, to detect very low concentrations of NO (2 ppb).

The above reported results demonstrate that the reduction route plays an important role in the gas sensing performances. GO flakes, reduced with p-phenylenediamine and deposited between metal electrodes to monitor their conductance, are more sensitive to dimethyl methylphosphonate (DMMP) than hydrazine reduced GO flakes. The sensing tests, performed at RT and in humid environment (RH < 20%), show that the device can detect 5 ppm of DMMP with recovery times lower than the one recorded for other materials [125].

**Figure 12:**
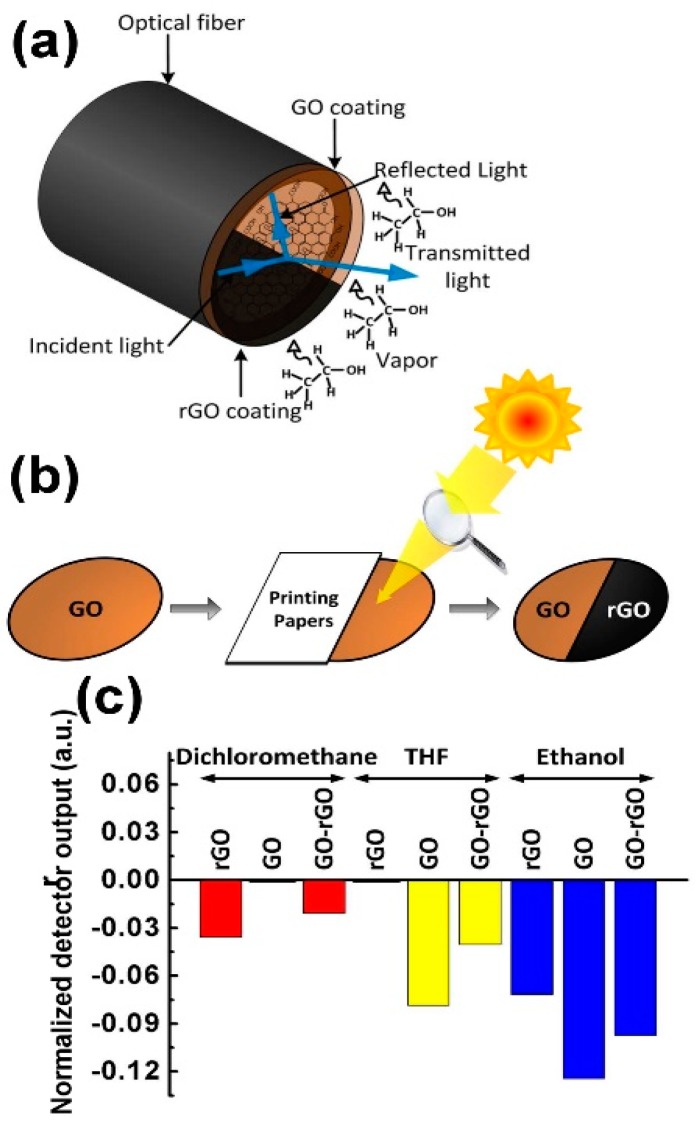
Schematic representation of the one-headed POF sensor covered with GO-rGO (**a**); Panel (**b**): fabrication process of the GO-rGO POF sensor by converting GO into rGO with sunlight; Panel (**c**): plot of the selectivity of one headed GO-rGO POF to THF, dichloromethane and ethanol. (Adapted with permission from Nature, Scientific Reports, London, United Kingdom, [86] copyright 2013).

To selectively detect VOCs, which is the main problem of GO- and rGO-based sensors, Some et al. proposed to deposit GO and rGO flakes (obtained by exposing GO flakes to sunlight) on a polymer optical fibre and to monitor its reflectance when exposed to different VOCs [86]. Due to the hydrophilicity of GO and the hydrophobicity of rGO flakes, some VOCs can adsorb on GO and cannot adsorb on the rGO and vice versa, changing (or not) the GO and rGO refractive indices. Combining the GO and rGO responses, the so-fabricated sensor array can distinguish between tetrahydrofuran (THF) and dichloromethane (MC). In Figure 12, the schematic representation of the fabrication procedure of the device and selectivity investigations are reported.

Functionalization and decoration of rGO flakes with metal nanostructure can have a beneficial effect on the sensing performances of the devices, in terms of response and selectivity. In particular, decoration and functionalization of rGO with metallic nanostructures has been investigated for the hydrogen sensing. Pt decorated holey rGO, constituting the semiconductive channel of a FET sensor [126], can detect H_2_ at RT, with a detection limit of 60 ppm. This device is selective to H_2_ over CO and CH_4_ and, more important, its response to H_2_ is not affected by humidity (with 11% ≤ RH ≤ 78%). Phan and Chung investigated the H_2_ sensing performances of a Pd nanocubes decorated rGO [127]. They found that its response is twice the one of a Pd NPs/rGO [128]. Furthermore, the device is selective to H_2_ over O_2_, NO_2_, CO and CO_2_ and can work at RT, even if the best performances are achieved at 50 °C. Finally, the same authors, in another work, show that the response increases with the increase of the Pd nanocubes size [129]. This explains the crucial role of the Pd nanostructures, due to their ability to dissociate the H_2_ molecules forming PdH_x_, which decreases the work function of Pd and allows the transfer of electrons to the rGO flakes and then to the electrodes. Optical gas sensors, with the sensitive layer constituted of Au NPs/rGO hybrid has been developed [46]. The absorbance of the Au NPs/rGO flakes is the sensing signal. The device can detect 100 ppm of H_2_ and 1 ppm of NO_2_ and it cannot detect CO (see Figure 5). In this work, the authors attribute the enhanced sensing performances of this device to the Localized Surface Plasmon Resonance (LSPR) of the Au NPs. A Ag NPs/rGO chemosensor can detect NH_3_ in dry air at RT [130]. The response to 10,000 ppm of NH_3_ is 17.4%, while the not-functionalized device shows a response of 0.2%. Furthermore, the response and recovery times (estimated as, respectively, the time required for the sensor signal to change from its value before the gas injection to the 63.2% of the final value during the gas injection and the time required for the sensor signal to recover the 63.2% of its value before the gas injection) are 6 s and 10 s. Also in this case, the metal NPs plays an important role in the gas sensing and Ag NPs are the dominant active adsorption sites for NH_3_. Ag NPs have been used also to decorate sulfonated rGO (S-rGO). The Ag-S-rGO has been used as sensitive layer for a chemiresistor sensor, to detect NO_2_ at RT, with 30% RH [131]. The response of the device to 0.5 ppm of NO_2_ is about 5% (estimated as the resistance relative change) and the response and recovery times for 50 ppm of NO_2_ are 12 s and 20 s. The authors found that the humidity does not affect the NO_2_ sensing response. The enhanced gas sensing responses are attributed to the –SO_3_H groups of the S-rGO and to the Ag NPs.

Coupling the sensing effects of rGO and metal oxide nanostructures has been proposed as a viable method to obtain more selective and sensitive devices. ZnO nanowires (NWs)-rGO layer has been deposited on an interdigitated Al_2_O_3_ substrate and exposed to NO_2_, H_2_ and CH_4_ [132]. The sensing responses have been recorded at various OTs and 250 °C is the best OT in terms of response to target gases. The gas sensing tests, performed at 40% RH, show that the response to NO_2_ of the ZnO NWs-rGO device (estimated as the relative resistance change, 680% for 5 ppm of NO_2_) is 40% higher than the only ZnO NWs. This indicates the importance of the rGO flakes in the gas sensing responses. Lower response to NO_2_ (25.6%) has been recorded at RT, in dry air environment, for a ZnO NPs-rGO device [133]. This resistive device has a limit of detection of 1 ppm. Other metal oxide nanostructures have been coupled to rGO to detect NO_2_ at RT. In_2_O_3_ cubes-rGO, constituting the active layer for a resistive gas sensor, can detect 1 ppm of NO_2_ at RT and 50% RH [134]. The response of the device to 5 ppm of NO_2_ is 60.80% and it is selective to 1 ppm of NO_2_ versus 1000 ppm of NH_3_, ethanol, acetone, H_2_ and CH_4_. In_2_O_3_ NPs-rGO, fabricated by hydrothermal method, also shows the ability to detect NO_2_ [135]. The environmental humidity can affect the sensing performances of metal oxide-rGO sensors. WO_3_-rGO nanocomposite films, used for fabrication of resistive sensors, can detect 0.5 ppm of NO_2_ at RT [136]. However, the response (calculated for 5 ppm of NO_2_) is about 900% for 30% RH and it reduces to 50% for 80% RH. This is very likely due to the effect of physisorbed H_2_O molecules, which occupy active sites, hindering the adsorption of NO_2_ molecules on WO_3_-rGO film. Very low detection limits of NO_2_ can be obtained in dry air or N_2_ atmosphere. Cu_2_O NWs-rGO resistive sensor has an estimated detection limit of 64 ppb [137], while ZnO nanorods-rGO can detect 47 ppb of NO_2_ [138]. Many authors highlight the fact that the p-n or p-p junction between MOX and rGO improves the response of the devices respect to the only MOX devices: the adsorbed gas molecules can influence the thickness of the depletion layer at the MOX-rGO interfaces. Furthermore, the rGO flakes constitute a preferential path for the charge carriers, improving the response and the sensing dynamics. For example, NiO nanosheets-rGO flakes resistive device show lower response and recovery times respect to NiO nanosheets devices, even if a complete recovery of the base line is not achieved at RT [139]. The NiO-rGO device is selective to NO_2_ versus CO, NH_3_, C_2_H_5_OH, HCHO and C_6_H_6_. Furthermore, the NiO-rGO device has a more stable response than NiO sensors. An almost perfect recovery of the base line has been achieved at OT = 50 °C and RH 25% for SnO_2_ NPs-rGO resistive sensor [140]. The device can detect 0.5 ppm of NO_2_, with a linear relation between NO_2_ concentration and response. SnO_2_ has been coupled, in form of quantum dots (QDs), to rGO to detect H_2_ and liquefied petroleum gas (LPG) [141]. The detection of these two gases with SnO_2_ QDs-rGO resistive sensors can be achieved at 200 °C and 250 °C OTs, respectively. This device shows the ability to detect H_2_ and LPG in standard humidity conditions (RH = 43%). The detection of H_2_ and LPG, at their relative OTs, is selective respect to 500 ppm of NH_3_, chloroform, toluene, benzene, acetone, N-butylacetate, acetic acid and formic acid. Other authors report lower OT for the detection of H_2_ with Pd-WO_3_-rGO heterostructures [142]. Pd-WO_3_ nanobelts-rGO flakes sensor, working at 100 °C, can detect H_2_ in a 20–10,000 ppm concentration range, in dry air. The sensing mechanism is influenced also by the presence of Pd, which can dissociate the hydrogen molecules. Hydrogen ions capture electrons from O^-^ ions on the WO_3_ surface, increasing the conductivity of the oxide. rGO flakes can modify the potential barrier at the rGO-WO_3_ interfaces, providing preferential pathways for carriers into the electrodes. As in the case of pure rGO and GO, engineering the surface of MOX-rGO can improve the sensing performances of the devices. Macroporous rGO-SnO_2_ NPs, -Fe_2_O_3_ NPs, -NiO NPs have been used for fabrication of resistive sensors [143]. Exposed to 200 ppm of ethanol in atmospheric air, at RT, the rGO-SnO_2_ device shows the highest response (about 55%, calculated as the ratio between the resistance before and during the gas injection) due to the p-n junction between the metal oxide and the rGO. Fe_2_O_3_ NPs have been used to detect ethanol at 280 °C OT and 50% RH [144]. Also in this case, rGO is important for the charge transport between electrodes and its presence increases the exposed sensing surface and the number of active sites, allowing to detect 1 ppm of ethanol. A selective and low detection limit resistive sensor of H_2_S has been reported, based on SnO_2_ quantum wire-rGO film [145]. This device can detect 43 ppb of H_2_S at RT and RH = 56–60%. The SnO_2_ quantum wire is the key sensing material, while rGO helps the transport of the charge carrier between the electrodes. The p-n heterojunction has been reported as responsible for enhancement of response of a resistive methanol sensor based on TiO_2_ nanotubes-rGO [146]. Also, the p-p homojunction between CuO nanoflowers and rGO can enhance the response of a resistive sensor to CO [147], even if the humidity affects the sensing performances of the device, which is quite selective to 50 ppm CO versus 50 ppm of CO_2_, H_2_, NO_2_, SO_2_, CH_4_ and NH_3_. Finally, some authors proposed the fabrication of a gas sensors array, constituted by a SnO_2_ nanospheres-rGO and CuO nanoflowers-rGO resistive sensors for the selective detection of NH_3_ and formaldehyde at RT [148].

Decorated rGO shows better sensing performances (in terms of selectivity, sensitivity and stability) than pristine GO or rGO. In particular, the heterojunctions forming at the MOX-rGO interfaces can enhance the sensing responses and the presence of rGO reduces the response and recovery times. The obtained results are promising, however high selectivity and full recovery of the base line are still unresolved tasks, especially at RT. The fabrication of GO and rGO composites, their functionalization with metal and metal oxide nanostructures, the implementation of different sensors in an array, can be viable solutions of these problems. UV illumination of the gas sensors for effective desorption of gas molecules adsorbed on the surface and therefore, to reduce the recovery times has been proposed for rGO-based sensors working at RT [117]. Furthermore, the above results show the advantage of using materials with high surface to volume ratio for gas sensing, suggesting using other 2D, semiconducting materials for gas detection.

## 4. MoS_2_ Gas Sensors

**Figure 13:**
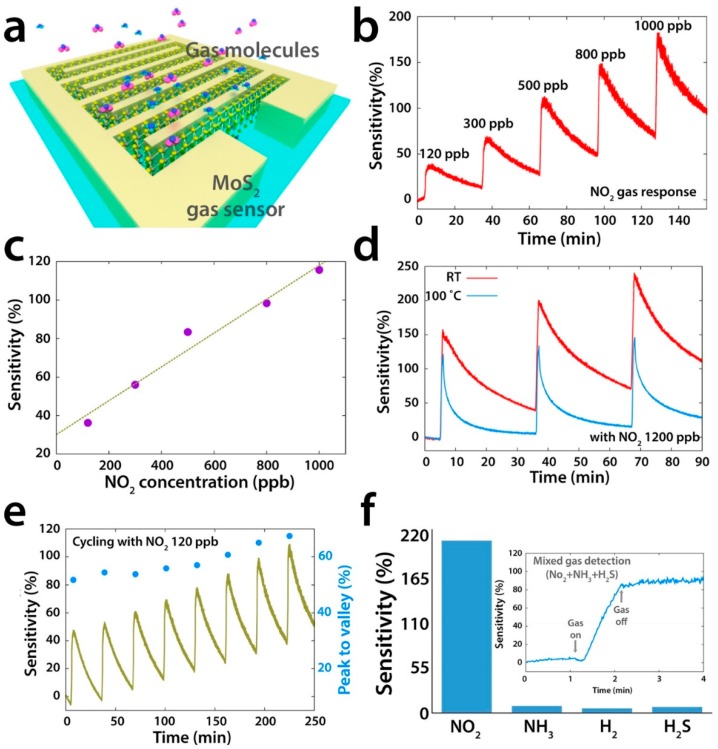
Panel (**a**): schematic image of the three-layers CVD grown MoS_2_ device in dark conditions; Panel (**b**): response of the MoS_2_ sensor to NO_2_ concentrations ranging from 120 ppb to 1 ppm; Panel (**c**): calibration curve of the MoS_2_ sensor; Panel (**d**): OT dependence of the response of the MoS_2_ sensor to 1200 ppb of NO_2_; Panel (**e**): reproducibility tests; Panel (**f**): results of the selectivity tests. (Reprintedwith permission from [161]. Copyright 2015 American Chemical Society, Washington, DC, USA).

MoS_2_, in its bulk form, is constituted of several S-Mo-S planes, bounded each other by weak van der Waals force. Due to this characteristic, as for graphite and its 2D counterpart graphene, MoS_2_ can be easily exfoliated, up to a monolayer, or it can be synthesized by chemical vapour deposition (CVD) [149]. The bulk MoS_2_ is an indirect bandgap semiconductor (1.2 eV), while monolayer MoS_2_ is a direct bandgap semiconductor (1.8 eV) [150]. The bulk MoS_2_ can be exfoliated by scotch tape, like graphene [15], or by lithium intercalation [151], or by sonication in a solvent with high surface tension, able to separate the layers [152,153]. The effective exfoliation up to monolayer and the way to count the number of layers of exfoliated or grown MoS_2_ flakes is based on Raman spectroscopy, due to the fact that Atomic Force Microscopy can overestimate the thickness of the flakes [154,155]. Differently from graphene, MoS_2_ is a semiconductor, with a bandgap ranging from 1.2 eV in bulk form (indirect) to 1.8 eV in monolayer phase (direct) [20,156,157]. The outstanding electronic properties of single layer MoS_2_ have become clear after the fabrication of the first transistor with micromechanically exfoliated MoS_2_ as the conductive channel [158]. Like in the case of graphene and graphene oxide, the high surface to volume ratio of mono and few layers MoS_2_ can be exploited for gas sensing applications. Theoretical calculations, based on Density Functional Theory (DFT), show that pollutant gases, like NO_2_, NO and SO_2_, can strongly interact with MoS_2_ surfaces [159,160]. Many experimental confirmations of these theoretical results have been reported. Resistive sensors based on 3 layers grown MoS_2_ has a NO_2_ detection limit, in dark conditions, of 120 ppb [161] (the sketch of this sensor and its gas sensing properties are reported in Figure 13).

**Figure 14:**
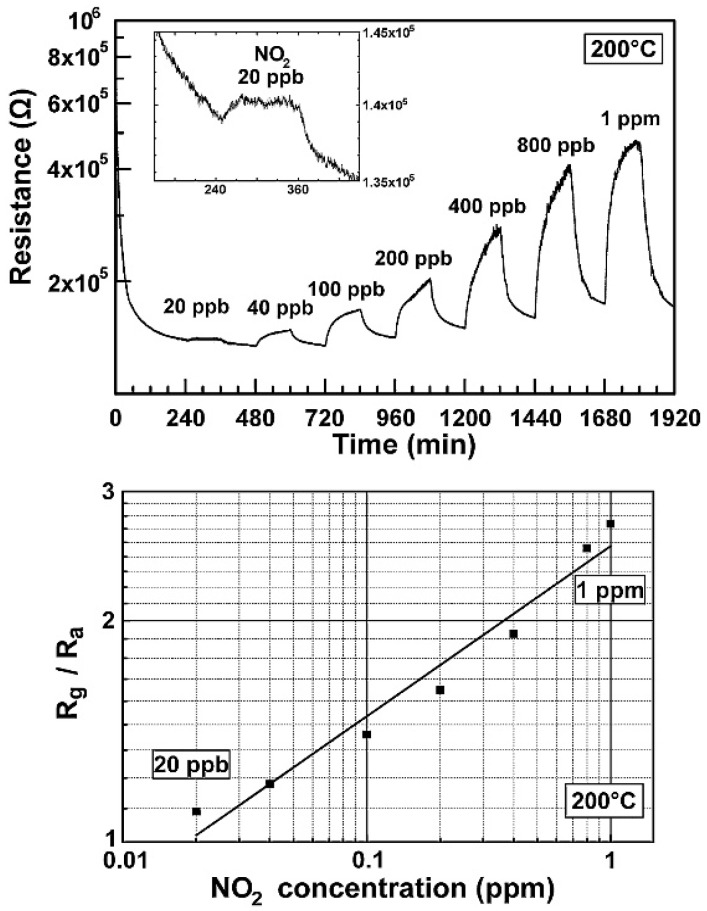
Top panel: dynamic response in dry air of the MoS_2_ device annealed at 250 °C to NO_2_ concentrations ranging from 20 ppb to 1 ppm, at OT = 200 °C; Bottom panel: calibration curve of the device. (Reprinted from [164], with permission from Elsevier, Amsterdam, The Netherlands).

The gas sensing measurements, performed in dry N_2_ atmosphere at RT, show the high selectivity of these n-type sensors to NO_2_ respect to other gases. When illuminated, the device is again sensitive to NO_2_ and NH_3_, however, the responses are lower than in the dark case. The authors ascribe this behaviour to the fact that illumination can accelerate the desorption of target gas molecules, which can be faster than the adsorption process. Monolayer MoS_2_, grown by CVD technique, has been used to fabricate FET-type gas sensors. In this configuration, tuning the gate voltage constitutes a way to selectively detect a target gas. Liu et al. reported that with a back gate voltage of 30 V, when the device is exposed to 400 ppb of NO_2_, the drain-source current strongly decreases, while, with no back gate voltage, when the device is exposed to 500 ppb of NH_3_, the drain-source current strongly increases [162]. The charge transfer mechanism between gas molecules and MoS_2_ has been proposed to explain the gas sensing properties of the device. If the device is exposed to NO_2_, which is an oxidizing gas, the electrons concentration in the MoS_2_ decreases and a more positive gate voltage has to be applied in order to switch on the n-type MoS_2_ FET. In the case of NH_3_, which is a reducing agent, the mechanism is the opposite, leading to low gate voltages to turn on the device. The measured detection limits are 20 ppb for the NO_2_ and 1 ppm for the NH_3_. FET MoS_2_ n-type devices has the ability to detect NO at RT in dry N_2_ atmosphere. In particular, it has been demonstrated that bi-layer MoS_2_ is more responsive than monolayer, with a NO detection limit of 0.3 ppm [163]. However, this device does not show a good recovery of the base line, which can be obtained increasing the OT or exposing the devices to UV or visible light. MoS_2_ powder has been exfoliated in N-methyl pyrrolidone in an ultrasonic bath, obtaining MoS_2_ flakes [164]. Then, the NMP/MoS_2_ flakes solution has been deposited on a pre-patterned substrate (with Pt electrodes on Si_3_N_4_ substrate, in an interdigitated configuration), in order to fabricate a resistive gas sensor. The so fabricated device has been exposed to NO_2_, showing good response at 150 °C and 200 °C OTs and a p-type behaviour, ascribed to residual solvent, doping the MoS_2_ surfaces. After thermal annealing in air at 250 °C, the MoS_2_-based device restores its usual n-type behaviour and, at 200 °C OT, it is sensitive to 20 ppb NO_2_. The dynamic response to NO_2_ in dry air and the calibration curve of the 250 °C annealed MoS_2_ device are reported in Figure 14.

**Figure 15:**
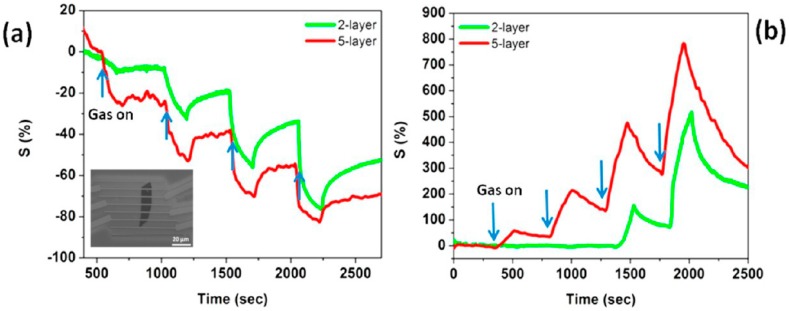
Sensing performances of 5-layers (red curve) and bilayers (green curve) MoS_2_ sensing device to NH_3_ (panel (**a**)) and NO_2_ (panel (**b**)). Gas concentrations are 100, 200, 500 and 1000 ppm. Inset: SEM image of the 2-layer MoS_2_ transistor device (scale bar 20 µm). (Adapted with permission from [166]. Copyright 2013 American Chemical Society, Washington, DC, USA).

Furthermore, both p-type and n-type devices are sensitive to RH changes. The flakes are not exfoliated up to monolayer, suggesting that there is no need of extreme exfoliation to obtain high sensing responses. MoS_2_ exfoliated flakes can be obtained also by sonication in chloroform and acetonitrile. The MoS_2_ flakes size can be decreased increasing the sonication time. The solution, deposited on a pre-patterned substrate, shows the ability to detect RH changes, and, furthermore, decreasing the size of the flakes leads to a decrease of the response and recovery times. The resistance of the flakes decreases with increasing RH values, usual for n-type semiconductors [165]. Mechanically exfoliated MoS_2_ flakes have been used to fabricate FET devices for gas sensing [166]. The sensing properties of different flakes with different heights have been studied, at RT in dry N_2_ environment. The FETs show n-type conductance and are able to detect 100–1000 ppm of NO_2_ or NH_3_. The authors found that the multilayer device show higher sensing responses than the bilayer one, as can be seen in Figure 15.

**Figure 16:**
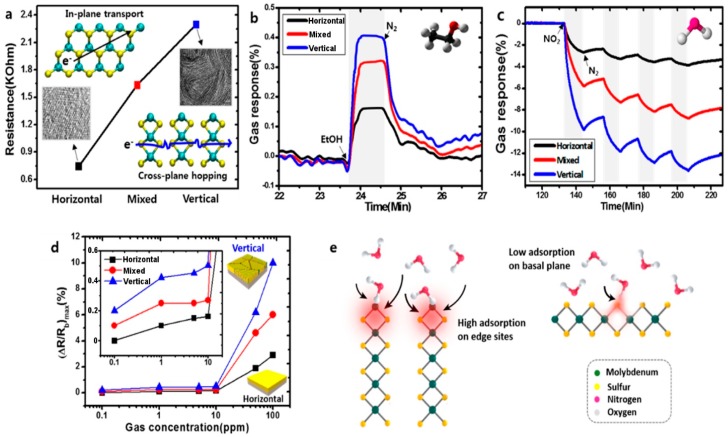
Panel (**a**): vertically aligned MoS_2_ flakes have higher resistance due to cross-plane hopping of the carriers; Panel (**b**): resistance change to 1000 ppm of ethanol for horizontally (black curve), vertically (blue curve) and mixed aligned MoS_2_ flakes; Panel (**c**): resistance change to 100 ppm of NO_2_ for horizontally (black curve), vertically (blue curve) and mixed aligned MoS_2_ flakes; Panel (**d**): relative resistance change of the horizontally, vertically and mixed aligned MoS_2_ flakes to 0.1–100 ppm NO_2_; Panel (**e**): schematic representation of the adsorption of NO_2_ molecules on edge sites and basal plane of the MoS_2_ flakes. (Reproduced with permission from [173]. Copyright 2015 American Chemical Society, Washington, DC, USA).

Furthermore, if a positive gate voltage is applied, the gas sensor increases its sensitivity to NO_2_, while decreases it to NH_3_. Scotch-tape exfoliated MoS_2_ flakes have been used for fabrication of resistive gas sensors, working at RT in dry N_2_ environment [167]. A monolayer exfoliated flake, deposited on SiO_2_/Si substrates and contacted by Au electrodes, shows high n-type responses to trimethylamine (with 10 ppb detection limit) and acetone (even if with a scarce recovery of the base line). The authors proposed a mechanism for the sensing: the Mo 3d_yz_ and S 2p orbitals extend over the MoS_2_ surface and are able to interact with the gas target molecules. The Mo 3d_yz_ orbitals are compensated by the Si orbitals of the substrate, while the positively charged S 2p orbitals are available for gas interaction, in particular with the donor-like analytes. Other exfoliation technique, like the above mentioned Li intercalation, can cause a change in the electronic and morphological properties of MoS_2_, changing its phase from the usual 2H to 1T and from semiconductive to metallic nature. The semiconductive MoS_2_ properties and its 2H phase can be recovered but the lithium intercalation can result in not reversible effects. Indeed, exfoliated MoS_2_ flakes, obtained by lithium intercalation and deposited between rGO electrodes over a flexible PET substrate, show p-type sensing behaviour [168]. The transistor, exposed to 1.2 ppm of NO_2_ in N_2_ environment, increases its drain-source current, reaching its maximum when the thickness of the MoS_2_ layer is 4 nm but it does not recover the base line after the NO_2_ is switched off. A slow but effective recovery of the base line can be obtained if the MoS_2_ flakes are decorated with Pt NPs, which also help to lower the NO_2_ detection limit to 0.5 ppm. A fast humidity sensor based on bulk-like MoS_2_ has been fabricated. The n-type MoS_2_ film has been deposited by dc magnetron sputtering on a p-type Si substrate, at RT and at 400 °C, forming a n-p junction. The device grown at 400 °C can detect RH changes at RT [169]. Lee et al. demonstrated the ability of vapour-phase grown MoS_2_ to detect NH_3_ in N_2_ environment at RT, with concentration higher than 2 ppm. Below that limit, the recovery of the base line is poor [170]. DFT calculations show the potential of MoS_2_ exfoliated flakes to adsorb H_2_ molecules, which prefer to bind with the S atoms of the monolayer, increasing its conductivity. If the MoS_2_ flakes are strained, the hydrogen molecules can be confined in the middle of the hexagon formed by S and Mo atoms, without the possibility to filter through the monolayer, due to the high energy barrier [171]. For non-polar gas molecules, like CO_2_ and CH_4_, the perfect MoS_2_ surface cannot offer adsorbing sites. The presence of defects, especially S vacancies, results in the possibility for CO_2_ and CH_4_ molecules to adsorb on MoS_2_ [172]. Furthermore, it has been demonstrated, by DFT calculations, that the edges of the MoS_2_ exfoliated flakes are more reactive and can constitute gas adsorption sites. The edge sites are therefore more active than the basal plane, leading to the fact that the flakes orientation can increase the response of the MoS_2_ flakes to target gases. Horizontally and vertically aligned MoS_2_ flakes have been fabricated by CVD. The NO_2_ response (in N_2_ atmosphere, at RT) of vertically aligned MoS_2_ flakes is five times higher than the horizontally aligned ones [173]. A comparison of the gas sensing performances of vertically and horizontally aligned MoS_2_ flakes is reported in Figure 16.

**Figure 17:**
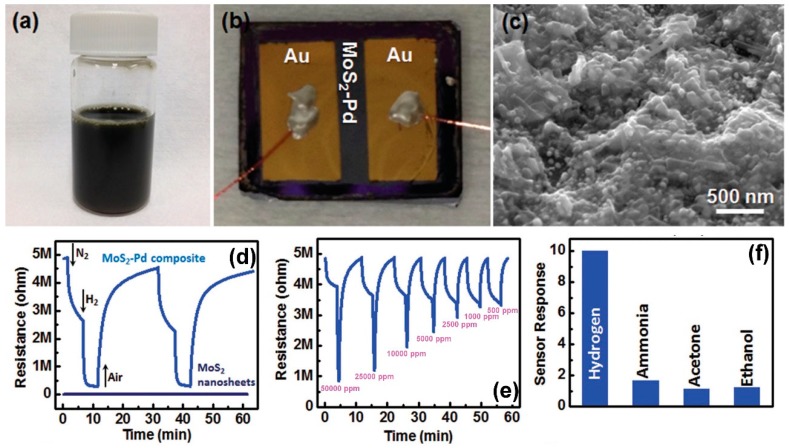
Panel (**a**): the MoS_2_-PdCl_2_ solution; Panel (**b**): MoS_2_-Pd FET, with gold electrodes; Panel (**c**): SEM image of the MoS_2_-Pd composite; Panel (**d**): comparison between the electrical responses of pristine MoS_2_ and Pd-MoS_2_ nanosheets to 50,000 ppm of H_2_; Panel (**e**): electrical responses of the Pd-MoS_2_ sensor exposed to H_2_ concentrations ranging from 50,000 to 500 ppm; Panel (**f**): selectivity of the Pd-MoS_2_ device to different target gases. (Adapted with permission from [182]. Published by Wiley-VCH Verlag GmbH & Co. KGaA, Weinheim, Germany).

As for the previously discussed GO and rGO, MoS_2_ flakes can be functionalize, in order to obtain more stable and more selective gas sensors. MoS_2_ flakes and nanostructures can be functionalized with metal oxide, like SnO_2_ and ZnO. Dispersed SnO_2_ NPs on MoS_2_ nanosheets can be obtain via hydrothermal methods [174]. The SnO_2_@MoS_2_ heterostructures are deposited on a patterned substrate, to perform gas sensing measurements at OTs of the order of hundreds of degrees, in dry air. The functionalized MoS_2_ shows higher ethanol sensing response than a SnO_2_ sensor and the optimal OT is lower (280 °C for the functionalized MoS_2_ sensor, 340 °C for SnO_2_-based one). Furthermore, the functionalized MoS_2_ sensor is selective to ethanol, respect to NH_3_, formaldehyde and acetone. Furthermore, SnO_2_ nanocrystals decoration can stabilize the MoS_2_ nanosheets, to obtain stable resistive devices working at RT [175]. Indeed, one of the main problems in gas sensing in standard conditions with MoS_2_ is that MoS_2_ current is not very stable and tends to drift in time. SnO_2_ decoration can solve this problem. The SnO_2_ decoration can also change the sensing behaviour of MoS_2_, from the usual n-type to p-type, due to the doping effect of SnO_2_ nanocrystals. The decorated resistive sensor is selective to NO_2_ (detection limit 0.5 ppm) in dry air environment, respect to H_2_, CO, H_2_S and NH_3_. Other metal oxide NPs, like ZnO NPs, have been used to functionalize MoS_2_. A hydrothermal method has been used to obtain ZnO-coated MoS_2_ nanosheets, which are then deposited on a substrate with previously patterned electrodes, in order to fabricate a resistive device [176]. The NPs size is about 8 nm, while the MoS_2_ size is about 500 nm. The ZnO decorated MoS_2_ has a selective response to ethanol at 260 °C OT in dry air, respect to methanol, NH_3_, benzene and methylbenzene. Besides metal oxide, metal NPs have been used to decorate MoS_2_ flakes, like Au, Pt, Pd and so forth. 10–20 nm Au NPs-loaded MoS_2_ resistive gas sensor has been fabricated. The presence of Au NPs helps the NH_3_ sensing of MoS_2_ flakes, due to their catalytic properties and their ability to increase the probability of interaction of NH_3_ molecules with MoS_2_ flake [177]. As a result, NH_3_ concentration down to 25 ppm in dry air can be detected, at low OT (60 °C). Other authors report the synthesis of Au NPs-decorated MoS_2_ flakes: MoS_2_ exfoliated flakes, obtained by chemical method, are mixed with HAuCl_4_ and subsequently annealed [178]. The MoS_2_ flakes defects and edges are the active sites for the synthesis of Au NPs. The so-fabricated Au@MoS_2_ flakes have been deposited on SiO_2_ substrate, with previously patterned electrodes, obtaining a resistive device. Due to the presence of SiO_2_ substrate, the not functionalized MoS_2_ shows a p-type behaviour, while the Au NPs n-dope the MoS_2_, with an overall n-type sensing behaviour. The doping effect of Au NPs can be exploited to tune the sensing performance of MoS_2_ to various VOCs. In particular, the response to toluene and hexane is positive, like not-functionalized MoS_2_, while MoS_2_ and Au decorated MoS_2_ have opposite responses to oxygen functionalized VOCs, like ethanol and acetone. These characteristics can be used to fabricate MoS_2_ based gas sensors array which can distinguish between different VOCs. Widely used noble metals to functionalize sensing layers are Pt and Pd. Pt NPs have been deposited on mechanically exfoliated MoS_2_ flakes, which constitute the conductive channel for a FET sensing device. Pt NPs deposited on a monolayer MoS_2_ flake can lead to a shift of the on-off threshold of the FET of 137 V. The effect on the threshold of the Pt NPs decreases with increasing MoS_2_ flakes thickness. Therefore, the doping effect of Pt NPs can be used and tuned for gas sensing applications [179]. Thermal evaporated Pd NPs have been deposited on mechanically exfoliated MoS_2_ flakes, on SiO_2_/Si substrate and contacted by graphene electrodes. The resulting resistive device has been exposed to NH_3_ and NO_2_, diluted in dry air, at 150 °C OT. The Pd-MoS_2_ is more sensitive to NH_3_ than the pristine MoS_2_ flake, while it is quite insensitive to NO_2_. If Al NPs are deposited on MoS_2_, instead of Pd NPs, the response to NH_3_ is the same of pristine MoS_2_, while the NO_2_ response increases. Furthermore, if the device is bended, the gas sensing performances improve. These effects of metal NPs on MoS_2_ flakes can be explained in terms of electronic and chemical sensization [180]. The ability of Pd NPs to improve the hydrogen sensing performances of MoS_2_ exfoliated flakes is well known and reported. The sensing mechanism is based on the electron transfer from the Pd NPs to the MoS_2_ flake, holes-doping it. Exposed to H_2_, Pd turns into PdH_x_, with a work function lower than MoS_2_ and bare Pd, therefore electrons pass from PdH_x_ to MoS_2_, compensating the holes-doping and lowering the electrical resistance of the Pd-MoS_2_ flake. This mechanism has been reported for resistive [181] and FET gas sensors [182]. In the resistive case, a not-continuous layer of Pd has been deposited on drop-casted MoS_2_ flakes. Gas sensing tests, performed at RT in dry air, show that the device resistance decreases when it is to hydrogen. For 5 nm thick Pd layer, the device has a response (calculated as the relative resistance change) of −10, with a detection limit of 50 ppm. In the FET case (Figure 17), Pd NPs have been deposited on MoS_2_ chemically exfoliated flakes. The H_2_ sensing performance of Pd functionalized flakes are better than the pristine MoS_2_, investigated in dry N_2_ environment at RT. Indeed, a complete recovery of the baseline, after the H_2_ is switched off, is achieved, without heat or UV light. Furthermore, the authors show the selective response of the functionalized MoS_2_-based FET device to H_2_, respect to acetone and ethanol. The p-doping effect of Pd NPs on MoS_2_ is demonstrated by the shift to higher voltage of the gate threshold respect to the on-off threshold of pristine MoS_2_ FET.

**Figure 18:**
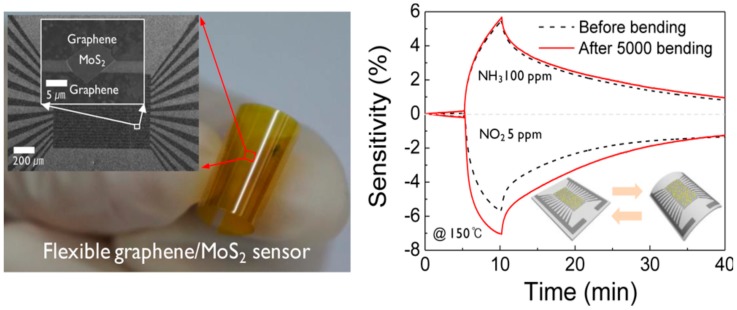
(**Left panel**) images of the MoS_2_-graphene heterostructure device; (**Right panel**) response of the device before and after bending to NO_2_ and NH_3_. (Reproduced with permission from [183]. Copyright 2015 American Chemical Society, Washington, DC, USA).

The obvious next step to the use of exfoliated MoS_2_ for gas sensing is to combine different 2D materials, in order to improve the sensing performances of the devices. Therefore, MoS_2_ has been coupled with graphene for the fabrication of gas sensors. Monolayer graphene has been CVD grown on mechanically exfoliated MoS_2_ flake and deposited between metal electrodes, obtaining a resistive gas sensor, as reported in Figure 18, left panel [183].

The device has good response to 1–5 ppm of NO_2_ and 5–100 ppm of NH_3_ in N_2_ atmosphere, at 150 °C OT. The OT is higher than for pristine MoS_2_ gas sensor, due to the defects in CVD grown graphene, which also improve the sensing responses. Furthermore, the sensing behaviour of the heterostructured device is p-type, differently from n-type pristine MoS_2_ gas sensors. The plastic substrate enables the bending of the device, which increases the gas sensing performances. Ultrasensitive resistive NO_2_ sensors (with an estimated detection limit of 5.7 ppb in air) have been fabricated, constituted of hydrothermally grown rGO/MoS_2_ heterostructures [184]. The created p-n junctions sensibly improve the gas sensing performances, indeed, the NO_2_ response of the rGO/MoS_2_ sensor is two times higher than rGO devices. The OT is 60 °C and the RH has a negligible effect on the sensing. The ratio between rGO and MoS_2_ (which can be estimated by the ratio between C and Mo) is an important factor in the gas sensing performances of rGO/MoS_2_ resistive devices. rGO enhances the charge transfer between MoS_2_ and target gases. rGO/MoS_2_ fibres, hydrothermally grown, used as sensing layer in a resistive gas sensor, have the best sensing response to NO_2_ and NH_3_ when C:Mo = 3:1. In this case, the limit of detection for NO_2_ at RT is 53 ppb [185]. Capping with other 2D nanostructure can also be useful to avoid the degradation of MoS_2_-based transistors or resistive gas sensors. h-BN exfoliated flakes capping MoS_2_ one, avoid the degradation of MoS_2_ and preserve the sensing capability of the devices [186].

The optical properties of MoS_2_ can be influenced by the adsorbed gas molecules on its surface. DFT calculations show that O_2_, NO_2_ and NO adsorbed molecules can change the dielectric constant of monolayer MoS_2_ [187]. Mechanically exfoliated monolayer MoS_2_ flake shows a photoluminescence (PL) peak at about 1.85 eV, when illuminated by 488 nm light source, recorded in a vacuum chamber. When O_2_, H_2_O and O_2_ + H_2_O are introduced inside the chamber, the PL signal increases its intensity of, respectively, 10, 35 and 100 times [188]. These results pave the way to the fabrication of gas sensor devices exploiting the optical properties of the mono and few layers MoS_2_.

The research on sensing properties of exfoliated MoS_2_ is developing during the last years and the MoS_2_ sensing performances are promising for the fabrication of low cost sensors. However, some problems are still unresolved: many of the reported MoS_2_ sensors work at OT higher than RT, increasing the power consumption of the device. Furthermore, the fabrication of single layer, high size MoS_2_ sheets is performed with bottom-up approaches, which can be expensive and not scalable. Another problem is related to the fact that the MoS_2_ basal plane does not have many adsorbing sites, differently from its edges, therefore, to obtain better sensing performances, the MoS_2_ flakes should be oriented. Further efforts should be devoted to the fabrication of a high number of exfoliated MoS_2_ flakes with high lateral size and to their functionalization, in order to obtain more selective and sensitive sensors, working at RT.

## 5. WS_2_ Gas Sensors

WS_2_ is a transition metal dichalcogenide, composed of several S-W-S layers, bounded each other by weak van der Waals forces. Similar to MoS_2_, due to the weak interaction between layers, it can be exfoliated up to monolayer. In this phase, each W atom is bounded to three S atoms, in a hexagonal configuration. Furthermore, similar to MoS_2_, WS_2_ is a semiconductor with an indirect bandgap in its bulk form (~1 eV) and a direct bandgap in its monolayer phase (~2 eV) [150]. Like for the other TMDs, in the last years, the researchers started to use and study WS_2_ as a sensing material, exploiting its electronic and morphological characteristics. Furthermore, theoretical studies, based on DFT + U calculations, show that adsorbed molecules on the monolayer WS_2_ can change the width of its bandgap [189]. NO and O_2_ molecules tend to withdraw more electrons from WS_2_ than H_2_O and CO, therefore a bigger charge transfer occurs. Also, ammonia molecules interact with WS_2_ monolayer and DFT calculations show that NH_3_ molecules act as electron donor for WS_2_, decreasing its work function, while the H_2_O molecules are electron acceptors and increase the WS_2_ work function [190]. A comparative study of the adsorption energy of various gas molecules on monolayer WS_2_ has been conducted [187]. The authors demonstrate that NO_2_ has the higher adsorption energy on WS_2_. Furthermore, NO_2_ and O_2_ molecules shift the transmission spectrum toward positive energies and lead to the emerging of an extra peak in the imaginary part of the dielectric constant of WS_2_. On the other hand, when the WS_2_ monolayer is exposed to NO molecules, the transmission spectrum shifts toward negative energies and the extra peak in the imaginary part of the dielectric constant appears at lower energies compared to O_2_ and NO_2_. All these studies highlight the fact that the adsorbed molecules on the mono and few layer WS_2_ can change its electronic and optical properties, giving new perspectives for its use in gas sensing, especially at RT. The effect of adsorbed water molecules on WS_2_ has been investigated, by performing resistance measurements of WS_2_ nanoparticles thin films (25–40 nm thick) fabricated by hot wire chemical vapour deposition (with a tungsten hot wire and H_2_S vapour) with varying RH values [191]. The current flowing into the WS_2_ sensing layer increases with increasing RH values, suggesting a p-type sensing behaviour of WS_2_ at RT. H_2_O adsorbed molecules influence also the optical properties of WS_2_. WS_2_ nanoflakes (size 20–200 nm) have been deposited on a side polished fibre (SPF), forming a 408 nm thick layer. Coated-SPF has been excited by a laser with a 1550 nm wavelength and the relative output optical power has been measured at different RH values. The relative output optical power varies between −6 dB (at RH = 35%) and −0.5 dB (at RH = 85%). The not-coated-SPF shows very little variations of the output optical power, indicating that its changes are due to the adsorption of water molecules on WS_2_. RH changes can be detected also studying the impedance change of WS_2_ flakes. Metallic WS_2_ flakes have been fabricated by t-Bu-Li intercalation. This exfoliation method causes the change of crystalline ordering of WS_2_, from semiconductive 2H to metallic 1T and can subsequently restored by thermal annealing. The impedance spectra of 1T-WS_2_ flakes, deposited on two electrodes, have been studied, with frequency varying in the 0.1 Hz–100 kHz range. Specific resonant frequencies have been found for methanol (around 1 Hz) and water vapour (around 1 kHz). The shift of the methanol specific resonant frequency can be used to obtain a calibration curve of the device, with methanol concentration lower than 100 ppm [45].

**Figure 19:**
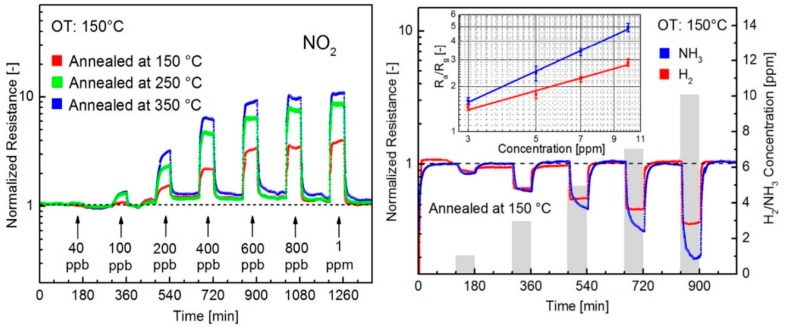
(**Left panel**) NO_2_ sensing responses of the WS_2_ sensors annealed at different temperatures; (**Right panel**) sensing responses to NH_3_ and H_2_ of the 150 °C annealed device. The inset reports the calibration curves for NH_3_ (blue curve) and H_2_ (red curve). (Adapted and reprinted from [193], with permission from Elsevier, Amsterdam, The Netherlands).

Above mentioned theoretical works report the ability of N-based molecules to adsorb on WS_2_ flakes. Sulfurized WO_3_ layers, resulting in WS_2_ thin films (2 nm–50 nm) can detect 1 ppm of NH_3_ in N_2_ at RT, even if a complete recovery of the baseline cannot be achieved [192]. Therefore, many authors fabricated WS_2_-based gas sensors to detect NO_2_ and NH_3_. Many of these sensors, like metal oxide gas sensors, do not work at RT, due to the fact that higher operating temperatures allow to obtain faster and complete recovery of the baseline. One of the main problem using OT different from RT is the partial oxidation of WS_2_. WS_2_ can be partially oxidized also at RT in WO_3_ amorphous form and a crystallization and nucleation process, leading to crystalline WO_3_, starts at 250 °C in air. Annealing WS_2_ at 150 °C in air results in the formation of a heterostructure of WS_2_/amorphous WO_3_, which shows better sensing performances than WS_2_ annealed at higher temperatures. The WS_2_ flakes (fabricated by liquid exfoliation and deposited on an interdigitated Si_3_N_4_ substrate to fabricate a resistive sensor and annealed at 150 °C in air) sensing characteristics have been analysed at OT = 150 °C. The device shows p-type sensing behaviour and outstanding detection limits in dry air for H_2_, NH_3_ and NO_2_ (1 ppm, 1 ppm and 100 ppb, respectively, Figure 19). Furthermore, 60% RH does not affect the sensing properties of the device, showing that it can be used in standard ambient conditions [193].

**Figure 20:**
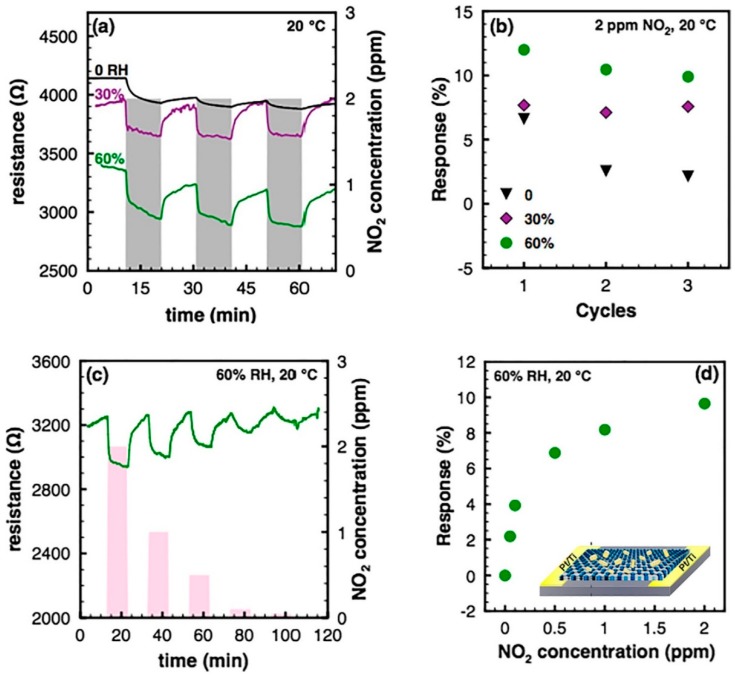
Panel (**a**): resistance change of the WS_2_/GA sensor to cyclic exposure to NO_2_, at different RH values; Panel (**b**): NO_2_ response of the device at different RH values; Panel (**c**): electrical response of the WS_2_/GA device to different concentrations of NO_2_ at RH = 60%; Panel (**d**): calibration curve of the device exposed to NO_2_ at RH = 60%. Inset: sketch of the resistive WS2/GA device. (Adapted and reprinted from [196], with permission from Elsevier, Amsterdam, The Netherlands).

Even lower detection limit to NO_2_ has been obtained by other authors with the fabrication of a conductometric gas sensor based on an aerogel composed of multiple stacked WS_2_ layers. They found that the device has a p-type behaviour and it can detect 8 ppb of NO_2_ at OT = 250 °C. The response time is of the order of few minutes. The presence of O_2_ molecules enhances the sensing properties of the material [194]. The selectivity of the gas sensing devices can be obtained performing the Principal Component Analysis (PCA) of the results. PCA has been used to distinguish between NO_2_, C_7_H_8_ and NH_3_ gas sensing signal of a resistive sensor based on a multitubular carbon nanofiber (MTCNF) functionalized by monolayer WS_2_ nanoflakes. The conjugated effect of WS_2_ and the edge rich structure and surface area of MTCNF allows to obtain a NO_2_ detection limit of 10 ppb (with a relative change of resistance of 0.29%) at RT in dry air, however, the presence of humidity reduces the response values [195]. In order to increase the selectivity of the gas sensors to a specific gas (in this case NO_2_), fabrication of heterostructure-based device has been proposed. Few layered WS_2_ flakes, coating a graphene aerogel (GA), have been used for the fabrication of a resistive device [196]. Graphene and WS_2_ have a p-type sensing behaviour and a potential barrier forms between them. NO_2_ molecules can take electrons from the GA, leading to a decrease of the potential barrier, while, for example, NH_3_ acts in the opposite way. At RT, the WS_2_/GA device can detect NO_2_, however, the recovery of baseline is very poor, due to the strong interaction between NO_2_ molecules and the heterostructure. OT = 180 °C is needed to obtain a complete recovery of the baseline. Furthermore, as observed in ref. [193], the humidity (with 0% < RH < 60%) does not affect the sensing response to NO_2_. The so-fabricated device, which is more selective to NO_2_ than its constituents (WS_2_ and GA), can detect a NO_2_ concentration of 10–15 ppb at OT = 180 °C. The sensing performances of this device are reported in Figure 20.

The not fully recovering of baseline constitutes a severe hurdle to the use of WS_2_-based gas sensors in everyday life. Atomic layer deposition (ALD) has been used to fabricate large area WS_2_ flakes and to have a control on their thickness [197]. Four-layers WS_2_ flakes, constituting the sensing layer for a resistive gas sensor operating at 100 °C, have the biggest response to NO_2_ compared to mono and bi-layer. However, the recovery of the baseline after the NO_2_ is switched off is poor. Therefore, the authors proposed to functionalize the WS_2_ flakes with Ag NWs. The functionalization decreases the conducibility of the nanostructure by two orders of magnitude, because the Ag NWs n-dope the WS_2_. However, the Ag functionalized WS_2_ has a response to NO_2_ twelve times higher than the pristine WS_2_ and a very good recovery of the baseline for NO_2_ concentrations in the 25–500 ppm range, with high selectivity to NO_2_. The Ag NWs and the WS_2_ edges act as adsorbing sites for NO_2_ molecules, which results in a cleavage of NO_2_ into NO and O. The WS_2_ functionalization with NPs is a viable way to obtain selectivity and high response to a certain gas. Pd NPs have been mixed in a solution containing previously ultrasonically-exfoliated WS_2_ flakes. After that, few drops of the solutions have been deposited on a flexible substrate, to obtain a flexible resistive gas sensor [198]. The device can detect H_2_ in N_2_ atmosphere at RT, decreasing its resistance when exposed to hydrogen gas (like a n-type semiconductor). As discussed for other Pd-functionalized 2D materials and metal oxides, Pd, after the adsorption of H_2_, becomes PdH_x_, decreasing its work function. The response of the device to 1000 ppm is 380% (estimated as the ratio between resistance before and during H_2_ exposure), higher than Pd-decorated carbon nanotubes and graphene. The H_2_ detection limit of the device is 10 ppm and the bending of the substrate does not affect the sensing performances. Another metal widely used to functionalize the sensing layers is Pt. Hydrothermally grown Pt quantum dots (QDs, size about 5 nm) have been dispersed in a solution containing WS_2_ nanosheets (1–10 nm thick), obtained by Li intercalation technique. Few drops of the solution have been deposited on a pre-patterned substrate, to fabricate a conductometric gas sensor, working at RT in dry air [199]. The sensor, whose sensing layer is a film of Pt QDs and WS_2_ nanosheets, with a Pt:W ratio of 0.1, has a response (calculated as the relative current change) of 3–20% in a range of 50–750 ppm of NH_3_ concentration. The presence of NH_3_ decreases the conductivity of the device and the Pt QDs enhance the sensing performance of the WS_2_, increasing its response to NH_3_ and making the device selective to ammonia. The interaction between Pt QDs and WS_2_ is crucial for the sensing mechanism: Pt has a Fermi level higher than WS_2_, therefore electrons are transferred from Pt to WS_2_, creating a hole depletion layer on the WS_2_ surface. NH_3_ molecules, at RT, interact with the oxygen species on the WS_2_ surface, releasing electrons, which are injected in the WS_2_, decreasing the conductivity. Furthermore, the presence on the WS_2_ surface of the Pt QDs increases the adsorbing sites for NH_3_ molecules. Beside functionalization with metal nanostructures, also metal oxide nanostructure can be exploited to obtain more selective and higher sensing responses of WS_2_. TiO_2_ QDs (size few nm) have been used to functionalize few layers WS_2_ sheets, obtaining a good reproducibility of the NH_3_ sensing response and good recovery of the baseline at RT in dry air [200]. The TiO_2_ functionalized WS_2_ has a response to NH_3_ 17 times higher than its pristine form and shows high selectivity to ammonia. Furthermore, TiO_2_ QDs dope the WS_2_ nanosheets, changing the usual p-type sensing behaviour to a n-type one. The sensing mechanism is similar to the one described before but in this case the NH_3_ presence causes an increase of the current flowing in the functionalized nanosheets. The presence of light illuminating the device can influence its gas sensing properties. A micromechanically exfoliated multi-layered flake of WS_2_ has been deposited on a SiO_2_/Si substrate and contacted with metal electrodes in FET configuration [201]. The I_ds_-V_g_ curves show that the WS_2_ flake has a n-type conductivity. The device is illuminated (wavelength 633 nm) and has the ability to detect O_2_, ethanol and NH_3_ gases, monitoring the changes of its external quantum efficiency (EQE). O_2_ molecules, acting as p-dopants, decreases the EQE, while reducing gases (NH_3_, ethanol), acting as n-dopants, increase the EQE. Monitoring the I_ds_ values, it is shown that the response to NH_3_ and ethanol is higher in dark conditions, while it is lower for O_2_.

WS_2_ exfoliated flakes show good sensing properties, in particular for the detection of NO_2_. Some works demonstrate that the environmental humidity does not affect the NO_2_ sensing, which is an important characteristic for the fabrication of effective gas sensors, used in everyday life. However, the best sensing performances (especially the full recovery of the base line) are achieved at temperatures of the order of hundreds °C, with the possible formation of WO_3_ and an increase of power consumption. The functionalization of WS_2_ exfoliated flakes can be a viable method to obtain selective and RT-working gas sensors.

## 6. Phosphorene Gas Sensors

**Figure 21:**
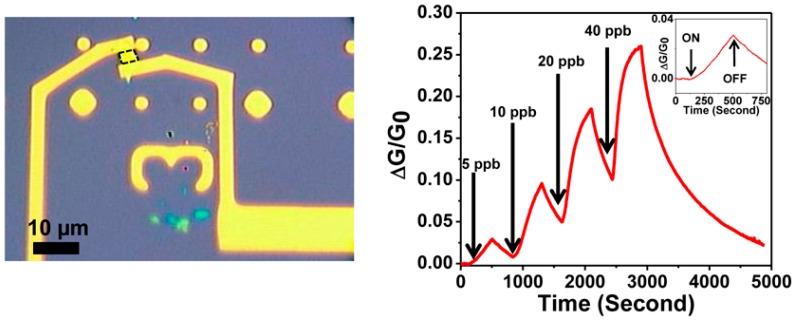
(**Left panel**) optical image of the phosphorene-based FET. The phosphorene flake is bounded by a dotted black line; (**Right panel**) conductance change of the phosphorene flakes exposed to different NO_2_ concentrations. (Reproduced with permission from [204]. Copyright 2015 American Chemical Society, Washington, DC, USA).

Phosphorene is the monolayer counterpart of black phosphorus (BP). It has a honeycomb structure, high carrier mobility and a tunable bandgap, ranging from 0.3 eV in bulk form to about 1.9 eV in monolayer [202] and it shows a p-type conductivity [21]. After its discovery and isolation, few layer BP flakes have been used to fabricate FETs [203,204]. Like other 2D materials, a single layer phosphorene flake can be obtained by mechanical exfoliation with a scotch tape [21], or by liquid exfoliation [202,205]. These electronic and morphological characteristics and the ease of fabrication, suggests the use of phosphorene for novel gas sensors. DFT calculations have been performed to estimate the adsorbing energies and charge transfer of several gas molecules adsorbed on a monolayer phosphorene. When adsorbed on the phosphorene surface, CO, H_2_, H_2_O and NH_3_ molecules act as electron donors, while NO, NO_2_ and O_2_ act as electron acceptors [206]. A big charge transfer is reported for H_2_O, NH_3_, NO, NO_2_ and O_2_. In particular, NO_2_, NO and O_2_ induce in-gap states in the phosphorene bandgap, due to their open shells. NO_2_ has the strongest interaction with phosphorene among the studied molecules, due to the hybridization of its frontier orbitals with the 3p orbitals of phosphorus. Therefore, DFT calculations suggest that phosphorene can be a useful sensing layer for NO_2_ sensors. Other theoretical calculations confirm the physisorbed NO and NO_2_ strong interaction with phosphorene and indicate that NH_3_ reduces the current flowing in the phosphorene, while NO increases it, which is a fingerprint of the p-type nature of the material. Furthermore, the current reduction when the NH_3_ molecules are adsorbed on the phosphorene surface, is observed only in the armchair directions, while there is no change in the current flowing in the zigzag direction [207]. Other authors report theoretical calculations on the ability of phosphorene to detect SO_2_, which is one of the main decomposition products of SF_6_. Therefore, the detection of SF_6_ can be performed through the sensing of SO_2_, which easily adsorbs on phosphorene and overlaps with the electron density of phosphorene, with high electron transfer [208]. One of the main problem with phosphorene as active layer for gas sensing devices is its tendency to oxidize also at RT in ambient conditions [203,209]. Therefore, many of the reported sensing experiments have been performed in dry air or in inert atmosphere. Mechanically exfoliated multilayer BP flakes have been deposited on a SiO_2_/Si substrate and metal electrodes have been patterned on it, in order to fabricate a FET for sensing applications [204] (Figure 21, left panel). As expected from theoretical calculations, the device shows a p-type conductivity, with an increase of the conductivity when oxidizing gases (in this case NO_2_) is inserted in the test chamber (Figure 21, right panel). The authors demonstrate that a BP-based FET device can detect 5 ppm of NO_2_ in Ar atmosphere, with a relative conductance change of 2.9%. The response time of the device is of the order of few minutes, while the recovery time is higher (of the order of 30 min), maybe due to the strong adsorption of NO_2_ molecules on the BP surface.

**Figure 22:**
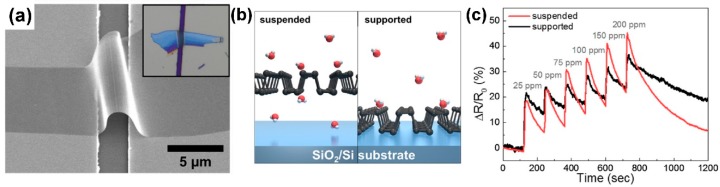
Panel (**a**): SEM image of the suspended phosphorene flake. Inset: optical image of the suspended flake; Panel (**b**): schematic illustration of the target gas molecules adsorbing on the suspended and supported phosphorene flake; Panel (**c**): responses of the suspended and the supported phosphorene flakes to increasing NO_2_ concentrations (from 25 to 200 ppm). (Adapted and reprinted from [211], with permission from Elsevier, Amsterdam, The Netherlands).

In a similar FET type configuration, the dependence of the sensing properties of mechanically exfoliated BP nanosheets to their heights have been studied [210]. The sensing tests have been performed in dry air at RT and they show that the sensing response to NO_2_ increases with the decrease of the flakes thickness, reaching its maximum for a 4.8 nm high flake and decreasing again for flakes with height < 4.8 nm. A 4.8 thick BP flake has a response of 190% (estimated as the relative conductance change) to 20 ppb of NO_2_. Increasing the BP exposed area can be a suitable way to obtain higher sensing response. In order to that, a mechanically exfoliated BP flake (20 nm high) has been suspended between two metal electrodes. The response, calculated as the relative resistance change, to 200 ppm of NO_2_ is 65%, 23% more than the response of a not-suspended flake [211] (Figure 22).

**Figure 23:**
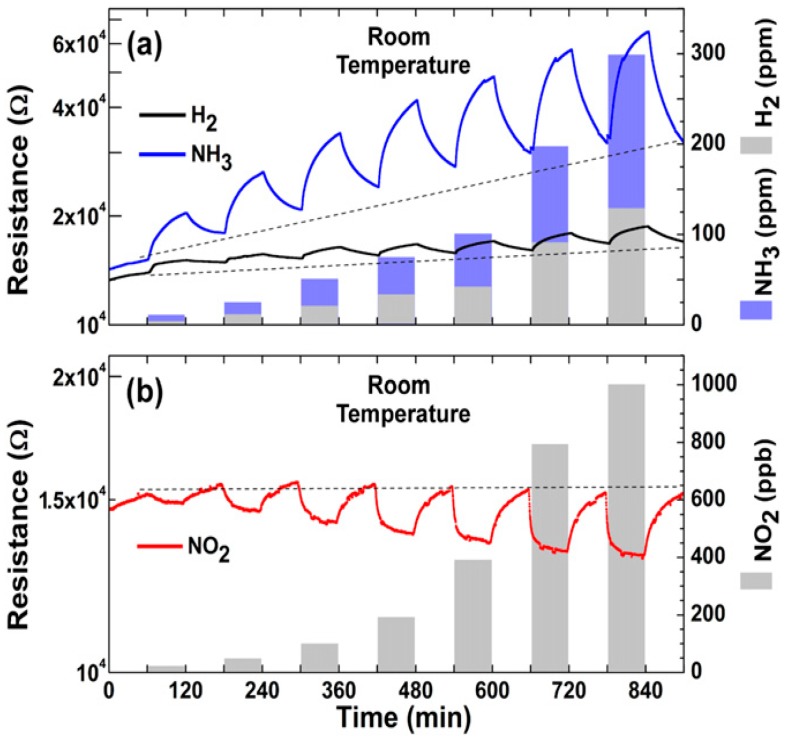
Panel (**a**): RT sensing responses of exfoliated BP flakes to H_2_ (black curve) and NH_3_ (blue curve); Panel (**b**): RT sensing responses of exfoliated BP flakes to NO_2_. (Reproduced with permission from [212], © IOP Publishing, Bristol, United Kingdom. Reproduced with all permission. All rights reserved).

To obtain a large number of exfoliated BP flakes, which can be subsequently deposited on an interdigitated and fabricate a chemiresistive device, liquid exfoliation, with the use of polar solvents (like NMP) and ultrasonication, has been proposed. A so-fabricated device has been used to detect NO_2_, NH_3_ and H_2_ in dry air, at RT [212]. The device electrical responses are reported in Figure 23. The BP flakes show a p-type conductivity, with an increase of resistance when exposed to ammonia and hydrogen and a decrease when exposed to NO_2_. The authors report that there is no response (as expected due to the low calculated adsorption energy and charge transfer) to CO and CO_2_. In 60 min, after the 60 min exposure to NO_2_, the recovery of the baseline is complete, while the NH_3_ molecules do not desorb completely from the BP surface. The estimated detection limits are 7 ppb (for NO_2_) and 1 ppm (for NH_3_), showing that liquid exfoliated BP flakes are promising for the fabrication of RT, easily to fabricate, gas sensing devices.

**Figure 24:**
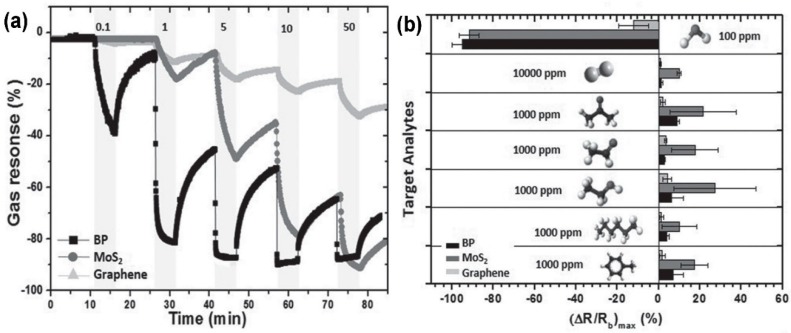
Panel (**a**): resistance variation of BP, MoS_2_ and graphene sensors exposed to 0.1, 1, 5, 10 and 50 ppm of NO_2_; Panel (**b**): the maximal resistance change onto various analytes of BP, MoS_2_ and graphene sensors. (Adapted and reprinted from [213], with permission from Wiley-VCH Verlag GmbH & Co. KGaA, Weinheim, Germany).

A comparison between liquid exfoliated BP, MoS_2_ and rGO flakes demonstrate the superior gas sensing properties of BP in N_2_ atmosphere and at RT, for the detection of NO_2_ [213]. Calculating the sensing response as the variation of the baseline resistance, the response to 1 ppm of NO_2_ is 10% for rGO, 15% for MoS_2_ and 80% for BP. Furthermore, BP can detect 0.1 ppm of NO_2_, while MoS_2_ and rGO do not. BP is 40 times faster than MoS_2_ and rGO to detect NO_2_ and it is more selective to NO_2_ respect to H_2_, acetone, acetyl aldehyde, ethanol, toluene and hexane, than the other investigated 2D materials. The main problem is that, after a storage in air for one month, BP is partly oxidized, even if there is not a sensible change in its responses to target gases. The gas sensing responses and selectivity of BP MoS_2_ and graphene are reported in Figure 24.

Liquid exfoliation of BP flakes has been performed also in ambient condition with N-cyclohexyl-2-pyrrolidone (CHP) [214]. The exfoliated flakes, after the deposition of metal electrodes, constitute the sensing layer for a resistive device, for the detection of NH_3_ in N_2_ atmosphere, at 10 Torr and at RT. The device increases its resistance when exposed to NH_3_ (p-type behaviour) and shows an estimated detection limit of 80 ppb. However, as in the case of ref. [212], the recovery of the baseline is poor. Impedance sensors, based on BP flakes, have been fabricated. Layered BP platelets have been fabricated by vapour transport growth from red phosphorus, which is another synthesis technique [215]. The BP platelets, constituting the active layer, are contacted by two metal electrodes and their impedances at RT have been monitored in the range of 0.1 Hz–100 kHz. A distinctive peak in the phase impedance spectra can be noticed at about 1 kHz when methanol is introduced in the test chamber. The phase is linearly dependent on the methanol concentration and it is very high selective versus air, toluene, acetone, chloroform, dichloromethane, ethanol, isopropanol and water [216]. The high capability of phosphorene to adsorb water molecules can be a problem because it facilitates the oxidation of BP, however, it can be very useful for the fabrication of humidity sensor working at RT. BP flakes, exfoliated by an electrochemical route, have been deposited on an interdigitated substrate, in FET configuration. The BP flakes show a p-type conductivity; however, the I_ds_ value increases with increasing RH values at RT [217]. This effect is contradictory with the p-type conductivity and the donor-like nature of H_2_O molecules. The sensing mechanism and the increase of I_ds_ is due to the “proton hopping” effect: the H_2_O molecules are adsorbed on the phosphorene surface and are divided into H^+^ and OH^−^ ions. Protons pass from one OH^−^ ions to another, forming H_3_O^+^. Therefore, the higher the RH value, the more effective is the “proton hopping” and the measured current is due to the protons movement. The increase of current flowing in the exfoliated BP flakes has been observed in many papers. Liquid exfoliated BP flakes have been filtered to obtain solutions with BP flakes with different thickness (about 80 nm, about 40 nm and few nm high). Then the flakes have been used as sensing layer for a humidity resistive device, working in N_2_ atmosphere at RT [218]. The response (calculated as the relative change of the resistance) is −40% and −65% for, respectively, 80 nm and 40 nm high flakes, with RH passing from 0 to 97.3%. For the few nm high flakes, the response is −92% with RH passing from 0 to 11.3%, reaching the −99% at RH = 57.57%, for higher RH values the response does not change a lot. Furthermore, the few nm high flakes device has a faster response (response time less than 5 min) and a recovery time of 10 s. Therefore, the thinnest flakes are more sensitive to RH changes. A liquid exfoliation method, based on the use of dimethylformamide (DMF) or dimethyl sulfoxide (DMSO), has been carried out to fabricate exfoliated BP flakes for the fabrication of a humidity resistive sensor at RT. This device works in the same way of the others described above, with a current increase with increasing RH [219]. The noticeable point is that the sensor does not show any changes in the response to RH after 3 months, being stable in ambient conditions and suggesting that the formation of phosphorus oxides is not detrimental for the humidity sensing. Other authors report that the oxidation of BP flakes leads to a decrease of the humidity sensing response of the BP-based sensors. Miao et al. [220], for example, have fabricated a transistor with a mechanically exfoliated flake as sensing material. In order to avoid the degradation of the BP flakes, a 6 nm thick Al_2_O_3_ layer has been deposited on the BP flakes and its sensing and conductivity properties have been compared with the ones of a not-capped BP flake. The Al_2_O_3_ capped BP flakes has an ambipolar conductivity, differently from the p-type conductivity of the pristine BP flake. The current increases when the RH values increase for both the devices. Furthermore, the humidity response of the pristine flake is higher than the capped one. However, after three days, the not-capped BP flake is not capable to detect humidity, while the capped one does not change its humidity response after 7 days. Liquid exfoliated BP flakes have been deposited on a QCM and its resonance frequency has been monitored at different RH values at RT [221]. The resonance frequency of the BP-covered QCM decreases with RH values increase. The response is reproducible and it is proportional to the amount of the deposited BP nanosheets.

DFT calculations have been carried out to define the capability of metal NPs functionalized phosphorene to detect CO, which cannot be detected by pristine phosphorene [222]. Pd-decorated phosphorene has the highest binding energy, therefore it can be used for CO detection, while Li, Na, K, Ca, Sr, Ba-phosphorene have lower binding energies and can be used for a reversible CO storage. Pt NPs (diameter about 3 nm) have been deposited on BP flakes (15–45 nm high) and arranged on a FET configuration. After that, the device has been covered with PMMA, to avoid the degradation of the flake [223]. The PMMA layer can stop O_2_, CO_2_ and H_2_O molecules, the main causes of the BP degradation but it let pass H_2_ molecules. The Pt-decorated BP flake has a p-type conductivity. Pristine BP is not able to detect H_2_ at RT, while Pt-functionalized BP can detect H_2_, due to the ability of Pt NPs to dissociate the H_2_ molecules, leading to a reduction of the Pt work function, to an electron transfer from Pt to BP and a current reduction, caused by the electron-hole recombination in the p-type BP flake. The recovery of the baseline is incomplete and very slow in N_2_ atmosphere, while it is accelerated by the presence of O_2_ molecules in dry air. Increasing the gate voltage to +40 V leads to an increase of the response and a decrease of the recovery time. The response (calculated as the relative difference of resistance of BP flake) is 15% for 6000 ppm of H_2_ in dry air. The ability of Pt-functionalized BP flakes to detect H_2_ has been observed also by other authors. Liquid exfoliated and Pt NPs decorated BP flakes have been used as sensing layer for a resistive device, showing a response (given by the relative difference of the resistance) of 500% for 1% H_2_ in N_2_ atmosphere at RT [224]. The authors demonstrate that Au NPs decoration of BP flakes change the conductivity type of the BP flakes (from p- to n-type), leading to an increase of the resistance when exposed to NO_2_ gas at RT in N_2_ atmosphere. Furthermore, the Au-decorated BP flakes sensor is selective to NO_2_ and is highly stable and has a low noise baseline.

Exfoliated BP flakes, used as sensing layer in chemiresistive devices, show better sensing properties (in particular for NO_2_ detection) than exfoliated MoS_2_ and graphene at RT. The recovery of the base line at RT can be achieved after tens of minutes, due to the strong adsorption of NO_2_ molecules on BP surface. Exfoliated BP-based gas sensors can operate at RT. However, the main problem of BP is its deterioration in humid atmosphere which affects the BP-based sensors stability. Capping the BP surface has been proposed to avoid degradation, however some target gases in this case cannot be detected. Also in this case, functionalization with metal NPs can result in selective gas sensors.

## 7. Conclusions

2D materials have gained a big interest in the last years in the gas sensing community. Their outstanding morphological and electronic properties have been widely investigated and their exploitation leads to the fabrication of many types of gas sensing devices, to detect many gases. The semiconductive 2D materials, in particular, join the morphological characteristics of graphene (in particular its extremely high surface to volume ratio) with their electronic properties, first of all their tunable bandgaps. The study on the functionalization of these materials is important to fabricate more selective devices. Metal NPs decoration has been widely used, especially for the detection of hydrogen. Furthermore, 2D materials show the ability to detect target gases even at RT, which is crucial to obtain low power consuming sensing devices.

In the Table 1, some of the most promising results for graphene oxide-, MoS_2_-, WS_2_-, exfoliated BP-based gas sensors are reported (in terms of limit of detection of target gases).

However, many problems still remain. First of all, the detection limits of the 2D materials-based device, now in the parts-per-billion range, should be decreased to parts-per-trillion. This goal can be achieved with the help of illumination (for example with UV lamps) and heating the device. On the other hand, these methods lead to an increase of the power consumption. Chemical modification of the surfaces, increasing the adsorbing sites, can be a viable method to decrease the detection limit and metal functionalization has been demonstrated to be useful to detect ppb concentration of target gases. The use of pre-concentrators, which can increase the concentration of the target gases and purify them from, for example, humidity, can push the detection limit of the gas sensors at the ppt limit. Humidity can strongly affect the sensing performances of the devices, leading to ambiguous responses to target gases. Therefore, humidity filters associated with the 2D materials-based sensors can overcome this problem.

Another issue is the slow recovery of the sensor baseline after the gas is switched off, in particular at RT. Gate voltage has been demonstrated to tune the recovery and response times and, therefore, FET type sensors can be designed and fabricated to work at RT, with low recovery times. Increasing the OTs is the usual way to obtain fast response and recovery and it is applied also for 2D materials-based sensors, with good results. For sensors working at RT, UV illumination can accelerate the desorption of gas molecules from the 2D materials surface and reduce the recovery times, and, with the LED technology, can be implemented in future commercial sensors. Very likely, the main obstacle to the commercialization of 2D materials-based gas sensors is their poor selectivity. In this review, we have reported many ways, basically based on the functionalization of the sensing layers, to obtain selective detection. For example, Pt and Pd functionalization of 2D materials surfaces has been widely used for selective detection of hydrogen molecules. Array of sensors based on different materials can be designed to obtain multivariable responses, whose analysis can lead to selectively detect one gas. Another way, not widely used, is to use PCA algorithms to distinguish between one gas or one compound from another. The deep understanding of the gas sensing mechanisms (with the help of theoretical calculations) can help to design and produce more selective 2D materials-based gas sensors. Water molecules, as the other target gas molecules, easily adsorb on the 2D materials surface and can deteriorate it. The effect of the humidity on the responses needs to be minimize. Also in this case, high OTs or humidity filters can help to achieve this goal. The response stability through months is a crucial point for the fabrication of everyday life sensors. 2D materials can degrade and oxidize in ambient air (phosphorene and WS_2_, for example, can oxidize at RT and their responses can change through days of operation). Therefore, capping the 2D materials with metal oxide, or polymers films, can prevent this effect.

Finally, another important point is the fabrication of these sensors. The fabrication costs of exfoliated flakes-based sensors can be low using liquid exfoliation methods and pre-patterned substrates. However, these methods can introduce contaminants, which should be carefully cleaned from the device. Mechanical exfoliation is less expensive but contacting the individual flakes (by EBL technique, for example), deposited in a completely random way on a substrate, is not a scalable process. Annealing in vacuum can remove contaminants from the surface of the 2D materials. The exfoliation routes and the fabrication methods of 2D materials-based sensors should be more investigated and carefully designed, in order to obtain more sensitive devices.

In conclusion, there is a lot of work to do, in order to fabricate gas sensors satisfying the 3S: sensitivity, selectivity and stability. In these last years, many researchers pave the way to the fabrication of 2D materials-based gas sensors, suggesting that they can substitute metal oxides gas sensing devices. 2D materials’ promising properties have been used, but, very likely, not fully exploited, for the scalable fabrication of reliable and low cost sensors.

## Figures and Tables

**Table 1:** A resume of the some of the devices reported in this paper with the lowest limit of detection.

Material	Device	Target Gas	LOD	OT (°C)	Notes	Ref.
GO	resistive	NO_2_	20 ppb in dry air	150	The responses for concentrations >40 ppb are not affected by RH	[81]
GO	resistive	NO_2_	650 ppb (est.)	RT	Sensing tests in dry air	[82]
GO	resistive	H_2_	100 ppm	RT	GO shows n-type behaviour. Low response and recovery times	[83]
edge-tailored GO	FET	SO_2_	5 ppm	RT	Sensing tests at 65% RH	[87]
fluorinated-GO	resistive	NH_3_	6 ppb (est.)	RT	Sensing tests in dry air	[94]
rGO	resistive	NH_3_	5 ppb	RT	Sensing tests at RH < 5%	[113]
holey rGO	resistive	NO_2_	60 ppb	RT	Sensing tests in dry air	[116]
rGO	resistive, flexible	NO_2_	400 ppt	RT	Sensing tests in dry air	[117]
rGO	resistive, flexible	NO_2_	50 ppb in dry air	RT	Sensing tests in ambient conditions show the ability to detect 1 ppm NO_2_	[118]
rGO-C nanodots	resistive	NO_2_	10 ppb	RT	Sensing tests in dry air. High selectivity to NO_2_	[121]
rGO	resistive	CO_2_	300 ppm	RT	Sensing tests in ambient conditions	[123]
Pd-RGO	resistive	NO	2 ppb	RT	Sensing tests in N_2_ atmosphere	[124]
Pt-rGO	FET	H_2_	60 ppm	RT	Sensing tests at 11% ≤ RH ≤ 78%. Selective to H_2_ over CO and CH_4_	[126]
Cu_2_O NWs-rGO	resistive	NO_2_	64 ppb (est.)	RT	Sensing tests in N_2_ atmosphere	[137]
ZnO nanorods-rGO	resistive	NO_2_	47 ppb (est.)	RT	Sensing tests in dry air	[138]
Pd-WO_3_ nanobelts-rGO	resistive	H_2_	20 ppm	100	Sensing tests in dry air. Good selectivity to H_2_. Recovery time (<1 min)	[142]
SnO_2_ quantum wire-rGO	resistive	H_2_S	43 ppb (est.)	RT	Sensing tests at RH = 56–60%	[145]
MoS_2_	resistive	NO_2_	120 ppb	RT	Sensing tests in N_2_ atmosphere	[161]
MoS_2_	FET	NO_2_	20 ppb	RT	Sensing tests in Ar atmosphere	[162]
MoS_2_	resistive	NH_3_	300 ppb	RT	Sensing tests in N_2_ atmosphere	[170]
Pd-MoS_2_	resistive	H_2_	50 ppm	RT	Sensing tests in dry air	[181]
rGO-MoS_2_	resistive	NO_2_	5.7 ppb (est.) in dry air	60	Selectivity to NO_2_ over NH3, H2S, CO and HCHO. Small humidity effects on response	[184]
rGO-MoS_2_ fibres	resistive	NO_2_	53 ppb (est.)	RT	Sensing tests in dry air	[185]
WS_2_	impedance	methanol	5.6 ppm (est.)	RT	Sensing tests in dry air	[45]
WS_2_	resistive	NO_2_	100 ppb in dry air	150	Partial oxidation of WS_2_ flakes. Humidity does not affect the sensing response	[193]
WS_2_	resistive	H_2_	1 ppm in dry air	150	Partial oxidation of WS_2_ flakes. Humidity does not affect the sensing response	[193]
WS_2_	resistive	NO_2_	8 ppb	250	Sensing tests in dry air	[194]
MTCNF-WS_2_	resistive	NO_2_	10 ppb	RT	Sensing tests in dry air. Humidity affects the sensing response	[195]
Pd NPs-WS_2_	resistive, flexible	H_2_	10 ppm	RT	Sensing tests in N_2_ atmosphere	[198]
Exfoliated BP	resistive	NO_2_	20 ppb	RT	Sensing tests in dry air	[210]
Exfoliated BP	resistive	NO_2_	7 ppb (est.)	RT	Sensing tests in dry air	[212]
Exfoliated BP	resistive	NH_3_	80 ppb (est.)	RT	Sensing tests in N_2_ atmosphere and at 10 Torr	[214]
Pt NPs- exfoliated BP	FET	H_2_	<2000 ppm (est.)	RT	Sensing tests in dry air. Pt-BP covered with PMMA. Selectivity to H2.	[223]

## References

[1] Janata J., Josowicz M. (2003). Conducting polymers in electronic chemical sensors. Nat. Mater..

[2] Miasik J.J., Hooper A., Tofield B.C. (1986). Conducting polymer gas sensors. J. Chem. Soc. Faraday Trans. 1.

[3] Virji S., Huang J., Kaner R.B., Weiller B.H. (2004). Polyaniline Nanofiber Gas Sensors: Examination of Response Mechanisms. Nano Lett..

[4] Li J., Lu Y., Ye Q., Cinke M., Han J., Meyyappan M. (2003). Carbon Nanotube Sensors for Gas and Organic Vapor Detection. Nano Lett..

[5] Wang Y., Yeow J.T.W. (2009). A Review on Carbon Nanotubes-Based Gas Sensors. J. Sens..

[6] Kanan S.M., El-Kadri O.M. (2009). Semiconducting Metal Oxide Based Sensors for Selective Gas Pollutant Detection. Sensors.

[7] Sun Y.-F., Liu S.-B., Meng F.-L., Liu J.-Y., Jin Z., Kong L.-T., Liu J.-H. (2012). Metal Oxide Nanostructures and Their Gas Sensing Properties: A Review. Sensors.

[8] Fine G.F., Cavanagh L.M., Afonja A., Binions R. (2010). Metal Oxide Semi-Conductor Gas Sensors in Environmental Monitoring. Sensors.

[9] Ponzoni A., Baratto C., Cattabiani N., Falasconi M., Galstyan V., Nunez-Carmona E., Rigoni F., Sberveglieri V., Zambotti G., Zappa D. (2017). Metal Oxide Gas Sensors, a Survey of Selectivity Issues Addressed at the SENSOR Lab, Brescia (Italy). Sensors.

[10] Wang C., Yin L., Zhang L., Xiang D., Gao R. (2010). Metal Oxide Gas Sensors: Sensitivity and Influencing Factors. Sensors.

[11] Bai H., Shi G. (2007). Gas Sensors Based on conducting Polymers. Sensors.

[12] Yoon H. (2013). Current Trends in Sensors Based on Conducting Polymer Nanomaterials. Nanomaterials.

[13] Cheah R., Forsyth M., Truong V.-T. (1998). Ordering and stability in conducting polypyrrole. Synth. Met..

[14] Wallace P.R. (1947). The Band Theory of Graphite. Phys. Rev..

[15] Novoselov K.S., Geim A.K., Morozov S.V., Jiang D., Zhang Y., Dubonos S.V., Grigorieva I.V., Firsov A.A. (2004). Electric Field Effect in Atomically Thin Carbon Films. Science.

[16] Weiss N.O., Zhou H., Liao L., Liu Y., Jiang S., Huang Y., Duan X. (2012). Graphene: An Emerging Electronic Material. Adv. Mater..

[17] Abergel D.S.L., Apalkov V., Berashevich J., Ziegler K., Chakraborty T. (2010). Properties of graphene: A theoretical perspective. Adv. Phys..

[18] Zhu H., Xu Z., Xie D., Fang Y. (2017). Graphene. Fabrication, Characterizations, Properties and Applications.

[19] Lin Y.-M., Avouris P. (2008). Strong Suppression of Electrical Noise in Bilayer Graphene Nanodevices. Nanoletters.

[20] Mak K.F., Lee C., Hone J., Shan J., Heinz T.F. (2010). Atomically Thin MoS_2_: A new direct-gap semiconductor. Phys. Rev. Lett..

[21] Liu H., Neal A.T., Zhu Z., Luo Z., Xu X., Tomanek D., Ye P.D. (2014). Phosphorene: An unexplored 2D semiconductor with a high hole mobility. ACS Nano.

[22] Choi W., Choudhary N., Han G.H., Park J., Akinwande D., Lee Y.H. (2017). Recent development of two-dimensional transition metal dichalcogenides and their applications. Mater. Today.

[23] Zappa D. (2017). Molybdenum Dichalcogenides for Environmental Chemical Sensing. Materials.

[24] Kim Y.-H., Phan D.-T., Ahn S., Nam K.-H., Park C.-M., Jeon K.-J. (2018). Two-dimensional SnS_2_ materials as high-performance NO_2_ sensors with fast response and high sensitivity. Sens. Actuators B-Chem..

[25] Mannix A.J., Kiraly B., Hersam M.C., Guisinger N.P. (2017). Synthesis and chemistry of elemental 2D materials. Nat. Rev. Chem..

[26] Kou L., Chen C., Smith S.C. (2015). Phosphorene: Fabrication, Properties, and Applications. J. Phys. Chem. Lett..

[27] Molle A., Grazianetti C., Tao L., Taneja D., Alam M.H., Akinwande D. (2018). Silicene, silicene derivatives, and their device applications. Chem. Soc. Rev..

[28] Vogt P., De Padova P., Quaresima C., Avila J., Frantzeskakis E., Asensio M.C., Resta A., Ealet B., Le Lay G. (2012). Silicene: Compelling Experimental Evidence for Graphenelike Two-Dimensional Silicon. Phys. Rev. Lett..

[29] Davila M.E., Xian L., Cahangirov S., Rubio A., Le Lay G. (2014). Germanene: A novel two-dimensional germanium allotrope akin to graphene and silicene. New J. Phys..

[30] Liu X., Ma T., Pinna N., Zhang J. (2017). Two-Dimensional Nanostructured Materials for Gas Sensing. Adv. Funct. Mater..

[31] Joshi N., Hayasaka T., Liu Y., Liu H., Oliveira O.N., Lin L. (2018). A review on chemiresistive room temperature gas sensors based on metal oxidenanostructures, graphene and 2D transition metal dichalcogenides. Microchim. Acta.

[32] Yang S., Jiang C., Wei S. (2017). Gas sensing in 2D materials. Appl. Phys. Rev..

[33] Varghese S.S., Varghese S.H., Swaminathan S., Singh K.K., Mittal V. (2015). Two-Dimensional Materials for Sensing: Graphene and Beyond. Electronics.

[34] Barsan N., Weimar U. (2001). Conduction Model of Metal Oxide Gas Sensors. J. Electroceram..

[35] Jiménez-Cadena G., Riu J., Rius F.X. (2007). Gas sensors based on nanostructured materials. Analyst.

[36] Leenaerts O., Partoens B., Peeters F.M. (2008). Adsorption of H_2_O, NH_3_, CO, NO_2_, and NO on graphene: A first –principles study. Phys. Rev. B.

[37] United States Environmental Protection Agency (2018). Primary National Ambient Air Quality Standards (NAAQS).

[38] Donarelli M., Milan R., Rigoni F., Drera G., Sangaletti L., Ponzoni A., Baratto C., Sberveglieri G., Comini E. (2018). Anomalous gas sensing behaviors to reducing agents of hydrothermally grown α-Fe_2_O_3_ nanorods. Sens. Actuators B-Chem..

[39] Gurlo A., Bârsan N., Oprea A., Sahm M., Sahm T., Weimar U. (2004). An n- to p-type conductivity transition induced by oxygen adsorption on α-Fe_2_O_3_. Appl. Phys. Lett..

[40] Arafat M.M., Dinan B., Akbar S.A., Haseeb A.S.M.A. (2012). Gas Sensors Based on One Dimensional Nanostructured Metal-Oxides: A Review. Sensors.

[41] Korotcenkov G., Cho B.K. (2011). Instability of metal oxide-based conductometric gas sensors and approaches to stability improvement (short survey). Sens. Actuators B-Chem..

[42] Wu Z., Chen X., Zhu S., Zhou Z., Yao Y., Quan W., Liu B. (2013). Enhanced sensitivity of ammonia sensor using graphene/polyaniline nanocomposite. Sens. Actuators B-Chem..

[43] Lu G., Park S., Yu K., Ruoff R.S., Ocola L.E., Rosenmann D. (2011). Toward Practical Gas Sensing with Highly Reduced Graphene Oxide: A New Signal Processing Method to Circumvent Run-to-Run and Device-to-Device Variations. ACS Nano.

[44] Barochi G., Rossignol J., Bouvet M. (2011). Development of microwave gas sensors. Sens. Actuators B-Chem..

[45] Mayorga-Martinez C.C., Ambrosi A., Eng A.Y.S., Sofer Z., Pumera M. (2015). Metallic 1T-WS_2_ for Selective Impedimetric Vapor Sensing. Adv. Funct. Mater..

[46] Cittadini M., Bersani M., Perrozzi F., Ottaviano L., Wlodarski W., Martucci A. (2014). Graphene oxide coupled with gold nanoparticles for localized surface plasmon resonance based gas sensor. Carbon.

[47] Zeng S., Baillargeat D., Ho H.-P., Yong K.-T. (2014). Nanomaterials enhanced surface plasmon resonance for biological and chemical sensing applications. Chem. Soc. Rev..

[48] Piliarik M., Homola J. (2009). Surface plasmon resonance (SPR) sensors: Approaching their limits?. Opt. Express.

[49] Zhang H., Sun Y., Gao S., Zhang J., Zhang H., Song D. (2013). A Novel Graphene Oxide-Based Surface Plasmon Resonance Biosensor for Immunoassey. Small.

[50] Zeng S., Hu S., Xia J., Anderson T., Dinh X.-Q., Meng X.-M., Coquet P., Yong K.-T. (2015). Graphene-MoS_2_ hybrid nanostructures enhanced surface plasmon resonances biosensors. Sens. Actuators B-Chem..

[51] Sauerbrey G. (1959). Verwendung von Schwingquarzen zur Wägung dünner Schichten und zur Microwägung. Z. Phys..

[52] Vashist S.K., Vashist P. (2011). Recent Advances in Quartz Crystal Microbalance-Based Sensors. J. Sens..

[53] Quang V.V., Hung V.N., Tuan L.A., Phan V.N., Huy T.Q., Quy N.V. (2014). Graphene-coated quartz crystal microbalance for detection of volatile organic compounds at room temperature. Thin Solid Films.

[54] Seekaew Y., Lokavee S., Phokharatkul D., Wisitsoraat A., Kerdcharoen T., Wongchoosuk C. (2014). Low-cost and flexible printed graphene-PEDOT:PSS gas sensor for ammonia detection. Org. Electron..

[55] Hong J., Lee S., Seo J., Pyo S., Kim J., Lee T. (2015). A Highly Sensitive Hydrogen Sensor with Gas Selectivity Using a PMMA Membrane-Coated Pd Nanoparticle/Single-Layer Graphene Hybrid. ACS Appl. Mater. Interfaces.

[56] Chung M.G., Kim D.-H., Seo D.K., Kim T., Im H.U., Lee H.M., Yoo J.-B., Hong S.-H., Kang T.J., Kim Y.H. (2012). Flexible hydrogen sensors using graphene with palladium nanoparticle decoration. Sens. Actuators B-Chem..

[57] Wu W., Liu Z., Jauregui L.A., Yu Q., Pillai R., Cao H., Bao J., Chen Y.P., Pei S.-S. (2010). Wafer-scale synthesis of graphene by chemical vapor deposition and its application in hydrogen sensing. Sens. Actuators B-Chem..

[58] Mu H., Zhang Z., Zhao X., Liu F., Wang K., Xie H. (2014). High sensitive formaldehyde graphene gas sensor modified by atomic layer deposition zinc oxide films. Appl. Phys. Lett..

[59] Yi J., Lee J.M., Park W.I. (2011). Vertically aligned ZnO nanorods and graphene hybrid architectures for high-sensitive flexible gas sensors. Sens. Actuators B-Chem..

[60] Yang Y., Tian C., Wang J., Sun L., Shi K., Zhou W., Fu H. (2014). Facile synthesis of novel 3D nanoflower-like Cu_x_O/multilayer graphene composites for room temperature NO_x_ gas sensor application. Nanoscale.

[61] Zhang Z., Zou R., Song G., Yu L., Chen Z., Hu J. (2011). Highly aligned SnO_2_ nanorods on graphene sheets for gas sensors. J. Mater. Chem..

[62] Hummers W.S., Offeman R.E. (1958). Preparation of Graphitic Oxide. J. Am. Chem. Soc..

[63] Daniela C., Marcano V.D., Kosynkin J.M., Berlin J.M., Sinitskii A., Sun Z., Slesarev A. (2010). Improved synthesis of graphene oxide. ACS Nano.

[64] Shen J., Hu Y., Shi M., Lu X., Qin C., Li C., Ye M. (2009). Fast and facile preparation of graphene oxide and reduced graphene oxide nanoplatelets. Chem. Mater..

[65] Zhang L., Liang J., Huang Y., Ma Y., Wang Y., Chen Y. (2009). Size-controlled synthesis of graphene oxide sheets on a large scale using chemical exfoliation. Carbon.

[66] Park S., Hu Y., Hwang J.O., Lee E.S., Casabianca L.B., Cai W., Potts J.R., Ha H.W., Chen S., Oh J. (2012). Chemical structures of hydrazine-treated graphene oxide and generation of aromatic nitrogen doping. Nat. Commun..

[67] Stankovich S., Dikin D.A., Piner R.D., Kohlhaas K.A., Kleinhammes A., Jia Y., Wu Y., Nguyen S.T., Ruoff R.S. (2007). Synthesis of graphene-based nanosheets via chemical reduction of exfoliated graphite oxide. Carbon.

[68] Treossi E., Melucci M., Liscio A., Gazzano M., Samorì P., Palermo V. (2009). High-Contrast Visualization of Graphene Oxide on Dye-Sensitized Glass, Quartz, and Silicon by Fluorescence Quenching. J. Am. Chem. Soc..

[69] Borini S., White R., Wei D., Astley M., Haque S., Spigone E., Harris N., Kivioja J., Ryhänen T. (2013). Ultrafast Graphene Oxide Humidity Sensors. ACS Nano.

[70] Huang X., Leng T., Georgiou T., Abraham J., Nair R.R., Novoselov K.S., Hu Z. (2018). Graphene Oxide Dielectric Permittivity at GHz and Its Applications for Wireless Humidity Sensing. Sci. Rep..

[71] Bi H., Yin K., Xie X., Ji J., Wan S., Sun L., Terrones M., Dresselhaus M.S. (2013). Ultrahigh humidity sensitivity of graphene oxide. Sci. Rep..

[72] Feng J., Kang X., Zuo Q., Yuan C., Wang W., Zhao Y., Zhu L., Lu H., Chen J. (2016). Fabrication and Evaluation of a Graphene Oxide-Based Capacitive Humidity Sensor. Sensors.

[73] Li N., Chen X.-D., Chen X.-P., Ding X., Li X.-Y. (2015). Subsecond Response of Humidity Sensor Based on Graphene Oxide Quantum Dots. IEEE Electr. Device Lett..

[74] Zhang K.-L., Hou Z.-L., Zhang B.-X., Zhao Q.-L. (2017). Highly sensitive humidity sensor based on graphene oxide foam. Appl. Phys. Lett..

[75] Feng X., Chen W., Yan L. (2015). Free-standing dried foam films of graphene oxide for humidity sensing. Sens. Actuators B-Chem..

[76] Yao Y., Chen X., Guo H., Wu Z., Li X. (2012). Humidity sensing behaviors of graphene oxide-silicon bi-layer flexible structure. Sens. Actuators B-Chem..

[77] Yao Y., Chen X., Guo H., Wu Z. (2011). Graphene oxide thin film coated quartz crystal microbalance for humidity detection. Appl. Surf. Sci..

[78] Yao Y., Chen X., Li X., Chen X., Li N. (2014). Investigation of the stability of QCM humidity sensor using graphene oxide as sensing films. Sens. Actuator. B-Chem..

[79] Chiu Y.-D., Wu C.-W., Chiang C.-C. (2017). Tilted Fiber Bragg Grating Sensor with Graphene Oxide Coating for Humidity Sensing. Sensors.

[80] Prezioso S., Perrozzi F., Giancaterini L., Cantalini C., Treossi E., Palermo V., Nardone M., Santucci S., Ottaviano L. (2013). Graphene Oxide as a Practical Solution to High Sensitivity Gas Sensing. J. Phys. Chem. C.

[81] Donarelli M., Prezioso S., Perrozzi F., Giancaterini L., Cantalini C., Treossi E., Palermo V., Santucci S., Ottaviano L. (2015). Graphene oxide for gas detection under standard humidity conditions. 2D Mater..

[82] Choi Y.R., Yoon Y.-G., Choi K.S., Kang J.H., Shim Y.-S., Kim Y.H., Chang H.J., Lee J.-H., Park C.R., Kim S.Y. (2015). Role of oxygen functional groups in graphene oxide for reversible room-temperature NO_2_ sensing. Carbon.

[83] Wang J., Singh B., Park J.-H., Rathi S., Lee I., Maeng S., Joh H.-I., Lee C.-H., Kim G.-H. (2014). Dielectrophoresis of graphene oxide nanostructures for hydrogen gas sensor at room temperature. Sens. Actuators B-Chem..

[84] Morales-Narváez E., Merkoçi A. (2012). Graphene Oxide as an Optical Biosensing Platform. Adv. Mater..

[85] Shavanova K., Bakakina Y., Burkova I., Shtepliuk I., Viter R., Ubelis A., Beni V., Starodub N., Yakimova R., Khranovskyy V. (2016). Application of 2D Non-Graphene Materials and 2D Oxide Nanostructures for Biosensing Technology. Sensors.

[86] Some S., Xu Y., Kim Y., Yoon Y., Qin H., Kulkarni A., Kim T., Lee H. (2013). Highly Sensitive and Selective Gas Sensor Using Hydrophilic and Hydrophobic Graphenes. Sci. Rep..

[87] Shen F., Wang D., Liu R., Pei X., Zhang T., Jin J. (2013). Edge-tailored graphene oxide nanosheet-based field effect transistors for fast and reversible electronic detection of sulfur dioxide. Nanoscale.

[88] Kolmakov A., Klenov D.O., Lilach Y., Stemmer S., Moskovitst M. (2005). Enhanced gas sensing by individual SnO_2_ nanowires and nanobelts functionalized with Pd catalyst particles. Nano Lett..

[89] Shin J., Choi S.-J., Lee I., Youn D.-Y., Park C.O., Lee J.-H., Tuller H.L., Kim I.D. (2013). Thin-wall assembled SnO_2_ fibers functionalized by catalytic Pt nanoparticles and their superior exhaled-breath-sensing properties for the diagnosis of diabetes. Adv. Funct. Mater..

[90] Guo J., Zhang J., Zhu M., Ju D., Xu H., Cao B. (2014). High-performance gas sensor based on ZnO nanowires functionalized by Au nanoparticles. Sens. Actuators B-Chem..

[91] Zhang Y., Xu J., Xu P., Zhu Y., Chen X., Yu W. (2010). Decoration of ZnO nanowires with Pt nanoparticles and their improved gas sensing and photocatalytic performance. Nanotechnology.

[92] Wang L., Wang S., Xu M., Hu X., Zhang H., Wang Y., Huang W. (2013). A Au-functionalized ZnO nanowire gas sensor for detection of benzene and toluene. Phys. Chem. Chem. Phys..

[93] Cattabiani N., Baratto C., Zappa D., Comini E., Donarelli M., Ferroni M., Ponzoni A., Faglia G. (2018). Tin Oxide Nanowires Decorated with Ag Nanoparticles for Visible Light-Enhanced Hydrogen Sensing at Room Temperature: Bridging Conductometric Gas Sensing and Plasmon-Driven Catalysis. J. Phys. Chem. C.

[94] Kim Y.H., Park J.S., Choi Y.-R., Park S.Y., Lee S.Y., Sohn W., Shim Y.-S., Lee J.-H., Park C.R., Choi Y.S. (2017). Chemically fluorinated graphene oxide for room temperature ammonia detection at ppb levels. J. Mater. Chem. A.

[95] Teradal N.L., Marx S., Morag A., Jelineka R. (2017). Porous graphene oxide chemi-capacitor vapor sensor array. J. Mater. Chem. C.

[96] Wang Z., Yang M., He J. (2014). Sensing Properties of GO and Amine-Silica Nanoparticles Functionalized QCM Sensors for Detection of Formaldehyde. Int. J. Nanosci..

[97] Stankovich S., Piner R.D., Chen X., Wu N., Nguyen S.T., Ruoff R.S. (2006). Stable aqueous dispersions of graphitic nanoplatelets via the reduction of exfoliated graphite oxide in the presence of poly(sodium 4-styrenesulfonate). J. Mater. Chem..

[98] Liu P., Gong K. (1999). Synthesis of polyaniline-intercalated graphite oxide by an in situ oxidative polymerization reaction. Carbon.

[99] Bourlinos A.B., Gournis D., Petridis D., Szabó T., Szeri A., Dékány I. (2003). Graphite Oxide:  Chemical Reduction to Graphite and Surface Modification with Primary Aliphatic Amines and Amino Acids. Langmuir.

[100] Gilje S., Han S., Wang M., Wang K.L., Kaner R.B. (2007). A Chemical Route to Graphene for Device Applications. Nano Lett..

[101] Gómez-Navarro C., Weitz R.T., Bittner A.M., Scolari M., Mews A., Burghard M., Kern K. (2007). Electronic Transport Properties of Individual Chemically Reduced Graphene Oxide Sheets. Nano Lett..

[102] Mattevi C., Eda G., Agnoli S., Miller S., Mkhoyan K.A., Mastrogiovanni D., Granozzi G., Garfunkel E., Chhowalla M. (2009). Evolution of Electrical, Chemical, and Structural Properties of Transparent and Conducting Chemically Derived Graphene Thin Films. Adv. Funct. Mater..

[103] Perrozzi F., Prezioso S., Donarelli M., Bisti F., De Marco P., Santucci S., Nardone M., Treossi E., Palermo V., Ottaviano L. (2013). Use of Optical Contrast to Estimate the Degree of Reduction of Graphene Oxide. J. Phys. Chem. C.

[104] Perrozzi F., Croce S., Treossi E., Palermo V., Santucci S., Fioravanti G., Ottaviano L. (2014). Reduction dependent wetting properties of graphene oxide. Carbon.

[105] Gilje S., Dubin S., Badakhshan A., Farrar J., Danczyk S.A., Kaner R.B. (2010). Photothermal Deoxygenation of Graphene Oxide for Patterning and Distributed Ignition Applications. Adv. Mater..

[106] Matsumoto Y., Koinuma M., Kim S.Y., Watanabe Y., Taniguchi T., Hatakeyama K., Tateishi H., Ida S. (2010). Simple Photoreduction of Graphene Oxide Nanosheet under Mild Conditions. ACS Appl. Mater. Interfaces.

[107] Cote L.J., Cruz-Silva R., Huang J. (2009). Flash Reduction and Patterning of Graphite Oxide and Its Polymer Composite. J. Am. Chem. Soc..

[108] Zhou Y., Bao Q., Varghese B., Ling Tang L.A., Tan C.K., Sow C., Loh K.P. (2010). Microstructuring of Graphene Oxide Nanosheets Using Direct Laser Writing. Adv. Mater..

[109] Zhang Y., Guo L., Wei S., He Y., Xia H., Chen Q., Sun H.-B., Xiao F.-S. (2010). Direct imprinting of microcircuits on graphene oxides film by femtosecond laser reduction. Nano Today.

[110] Prezioso S., Perrozzi F., Donarelli M., Bisti F., Santucci S., Palladino L., Nardone M., Treossi E., Palermo V., Ottaviano L. (2012). Large area extreme-UV lithography of graphene oxide via spatially resolved photoreduction. Langmuir.

[111] Prezioso S., Perrozzi F., Donarelli M., Stagnini E., Treossi E., Palermo V., Santucci S., Nardone M., Moras P., Ottaviano L. (2014). Dose and wavelength dependent study of graphene oxide photoreduction with VUV Synchrotron radiation. Carbon.

[112] Robinson J.T., Perkins F.K., Snow E.S., Wei Z., Sheenan P.E. (2008). Reduced Graphene Oxide Molecular Sensors. Nano Lett..

[113] Wang Y., Zhang L., Hu N., Wang Y., Zhang Y., Zhou Z., Liu Y., Shen S. (2014). Ammonia gas sensors based on chemically reduced graphene oxide sheets self-assembled on Au electrodes. Nanoscale Res. Lett..

[114] Ghosh R., Midya A., Santra S., Ray S.K., Guha P.K. (2013). Chemically Reduced Graphene Oxide for Ammonia Detection at Room Temperature. ACS Appl. Mater. Interfaces.

[115] Lu G., Ocola L.E., Chen J. (2009). Reduced graphene oxide for room-temperature gas sensors. Nanotechnology.

[116] Wang D.H., Hu Y., Zhao J.J., Zeng L.L., Tao X.M., Chen W. (2014). Holey reduced graphene oxide nanosheets for high performance room temperature gas sensing. J. Mater. Chem. A.

[117] Dua V., Surwade S.P., Ammu S., Agnihotra S.R., Jain S., Roberts K.E., Park S., Ruoff R.S., Manohar S.K. (2010). All-Organic Vapor Sensor Using Inkjet-Printed Reduced Graphene Oxide. Angew. Chem. Int. Ed..

[118] Duy L.T., Trung T.Q., Hanif A., Siddiqui S., Roh E., Lee W., Lee N.-E. (2017). A stretchable and highly sensitive chemical sensor using multilayered network of polyurethane nanofibres with self-assembled reduced graphene oxide. 2D Mater..

[119] Choi S.-J., Kim S.-J., Jang J.-S., Lee J.-H., Kim L.-D. (2016). Silver Nanowire Embedded Colorless Polyimide Heater for Wearable Chemical Sensors: Improved Reversible Reaction Kinetics of Optically Reduced Graphene Oxide. Small.

[120] Chen A., Liu R., Peng X., Chen Q., Wu J. (2017). 2D Hybrid Nanomaterials for Selective Detection of NO_2_ and SO_2_ Using “Light On and Off” Strategy. ACS Appl. Mater. Interfaces.

[121] Hu J., Zou C., Su Y., Li M., Hu N., Ni H., Yang Z., Zhang Y. (2017). Enhanced NO_2_ sensing performance of reduced graphene oxide by in situ anchoring carbon dots. J. Mater. Chem. C.

[122] Sridevi S., Vasub K.S., Bhat N., Asokan S., Sood A.K. (2016). Ultra sensitive NO_2_ gas detection using the reduced graphene oxide coated etched fiber Bragg gratings. Sens. Actuators B-Chem..

[123] Hafiz S.M., Ritikos R., Whitcher T.J., Razib N.M., Bien D.C.S., Chanlek N., Nakajima H., Saiposa T., Songsiriritthigul P., Huang N.M. (2014). A practical carbon dioxide gas sensor using room-temperature hydrogen plasma reduced graphene oxide. Sens. Actuators B-Chem..

[124] Li W., Geng X., Guo Y., Rong J., Gong Y., Wu L., Zhang X., Li P., Xu J., Cheng G. (2011). Reduced Graphene Oxide Electrically Contacted Graphene Sensor for Highly Sensitive Nitric Oxide Detection. ACS Nano.

[125] Hu N., Wang Y., Chai J., Gao R., Yang Z., Kong E.S.-W., Zhang Y. (2012). Gas sensor based on p-phenylenediamine reduced graphene oxide. Sens. Actuators B-Chem..

[126] Vedala H., Sorescu D.C., Kotchey G.P., Star A. (2011). Chemical Sensitivity of Graphene Edges Decorated with Metal Nanoparticles. Nano Lett..

[127] Phan D.-T., Chung G.-S. (2014). A novel Pd nanocube-graphene hybrid for hydrogen detection. Sens. Actuators B-Chem..

[128] Phan D.-T., Chung G.-S. (2014). Characteristics of resistivity-type hydrogen sensing based on palladium-graphene nanocomposites. Int. J. Hydrogen Energy.

[129] Phan D.-T., Chung G.-S. (2014). Effects of Pd nanocube size of Pd nanocube-graphene hybrid on hydrogen sensing properties. Sens. Actuators B-Chem..

[130] Cui S., Mao S., Wen Z., Chang J., Zhang Y., Chen J. (2013). Controllable synthesis of silver nanoparticle-decorated reduced graphene oxide hybrids for ammonia detection. Analyst.

[131] Huang L., Wang Z., Zhang J., Pu J., Lin Y., Xu S., Shen L., Chen Q., Shi W. (2014). Fully Printed, Rapid-Response Sensors Based on Chemically Modified Graphene for Detecting NO_2_ at Room Temperature. ACS Appl. Mater. Interfaces.

[132] Galstyan V., Comini E., Kholmanov I., Faglia G., Sberveglieri G. (2016). Reduced graphene oxide/ZnO nanocomposite for application in chemical gas sensors. RSC Adv..

[133] Liu S., Yu B., Zhang H., Fei T., Zhang T. (2014). Enhancing NO_2_ gas sensing performances at room temperature based on reduced graphene oxide-ZnO nanoparticles hybrids. Sens. Actuators B-Chem..

[134] Yang W., Wan P., Zhou X., Hu J., Guan Y., Feng L. (2014). Additive-Free Synthesis of In_2_O_3_ Cubes Embedded into Graphene Sheets and Their Enhanced NO_2_ Sensing Performances at Room Temperature. Appl. Mater. Interfaces.

[135] Gu F., Nie R., Han D., Wang Z. (2015). In_2_O_3_-graphene nanocomposite based gas sensor for selective detection of NO_2_ at room temperature. Sens. Actuators B-Chem..

[136] Su P.-G., Peng S.-L. (2015). Fabrication and NO_2_ gas-sensing properties of reduced graphene oxide/WO_3_ nanocomposite films. Talanta.

[137] Deng S., Tjoa V., Fan H.M., Tan H.R., Sayle D.C., Olivo M., Mhaisalkar S., Wei J., Sow C.H. (2012). Reduced Graphene Oxide Conjugated Cu_2_O Nanowire Mesocrystals for High-Perfomance NO_2_ Gas Sensor. J. Am. Chem. Soc..

[138] Xia Y., Wang J., Xu J.-L., Li X., Xie D., Xiang L., Komarneni S. (2016). Confined Formation of Ultrathin ZnO Nanorods/Reduced Graphene Oxide Mesoporous Nanocomposites for High-Performance Room-Temperature NO_2_ Sensors. ACS Appl. Mater. Interfaces.

[139] Zhang J., Zeng D., Zhao S., Wu J., Xu K., Zhu Q., Zhang G., Xie C. (2015). Room temperature NO_2_ sensing: What advantage does the rGO-NiO composite have over pristine NiO?. Phys. Chem. Chem. Phys..

[140] Zhang H., Feng J., Fei T., Liu S., Zhang T. (2014). SnO_2_ nanoparticles-reduced graphene oxide nanocomposites for NO_2_ sensing at low operating temperature. Sens. Actuators B-Chem..

[141] Mishra R.K., Upadhyay S.B., Kushwaha A., Kim T.-H., Murali G., Verma R., Srivastava M., Singh J., Sahay P.P., Lee S.H. (2015). SnO_2_ quantum dots decorated on RGO: A superior sensitive, selective and reproducible performance for a H_2_ and LPG sensor. Nanoscale.

[142] Esfandiar A., Irajizad A., Akhavan O., Ghasemi S., Gholami M.R. (2014). Pd-WO_3_/reduced graphene oxide hierarchical nanostructures as efficient hydrogen gas sensors. Int. J. Hydrogen Energy.

[143] Xu S., Sun F., Pan Z., Huang C., Yang S., Long J., Chen Y. (2016). Reduced Graphene Oxide-Based Ordered Macroporous Films on a Curved Surface: General Fabrication and Application in Gas Sensors. ACS Appl. Mater. Interfaces.

[144] Liang S., Zhu J., Wang C., Yu S., Bi H., Liu X., Wang X. (2014). Fabrication of α-Fe_2_O_3_@graphene nanostructures for enhanced gas-sensing property to ethanol. Appl. Surf. Sci..

[145] Song Z., Wei Z., Wang B., Luo Z., Xu S., Zhang W., Yu H., Li M., Huang Z., Zang J. (2016). Sensitive Room-Temperature H_2_S Gas Sensors Employing SnO_2_ Quantum Wire/Reduced Graphene Oxide Nanocomposites. Chem. Mater..

[146] Acharyya D., Bhattacharyya P. (2016). Highly Efficient Room-Temperature Gas Sensor Based on TiO_2_ Nanotube-Reduced Graphene-Oxide Hybrid Device. IEEE Electron Device Lett..

[147] Zhang D., Jiang C., Liu J., Cao Y. (2017). Carbon monoxide gas sensing at room temperature using copper oxide-decorated graphene hybrid nanocomposite prepared by layer-by-layer self-assembly. Sens. Actuators B-Chem..

[148] Zhang D., Liu J., Jiang C., Liu A., Xia B. (2017). Quantitative detection of formaldehyde and ammonia gas via metal oxide-modified graphene-based sensor array combining with neural network model. Sens. Actuators B-Chem..

[149] Li X., Zhu H. (2015). Two-dimensional MoS_2_: Properties, preparation, and applications. J. Mater..

[150] Wang Q.H., Kalantar-Zadeh K., Kis A., Coleman J.N., Strano M.S. (2012). Electronics and optoelectronics of two-dimensional transition metal dichalcogenides. Nat. Nanotechnol..

[151] Wan J., Lacey S.D., Dai J., Bao W., Fuhrer M.S., Hu L. (2016). Tuning two-dimensional nanomaterials by intercalation: Materials, properties and applications. Chem. Soc. Rev..

[152] Coleman J.N., Lotya M., O’Neill A., Bergin S.D., King P.J., Khan U., Young K., Gaucher A., De S., Smith R.J. (2011). Two-Dimensional Nanosheets Produced by Liquid Exfoliation of Layered Materials. Science.

[153] Zhou K., Mao N., Wang H., Peng Y., Zhang H. (2011). A Mixed-Solvent Strategy for Efficient Exfoliation of Inorganic Graphene Analogues. Angew. Chem..

[154] Li H., Zhang Q., Yap C.C.R., Tay B.K., Edwin T.H.T., Olivier A., Baillargeat D. (2012). From Bulk to Monolayer MoS_2_: Evolution of Raman Scattering. Adv. Funct. Mater..

[155] Ottaviano L., Palleschi S., Perrozzi F., D’Olimpio G., Priante F., Donarelli M., Nardone M., Benassi P., Gonchigsuren M., Gombosuren M. (2017). Mechanical exfoliation and layer number identification of MoS_2_ revisited. 2D Mater..

[156] Splendiani A., Sun L., Zhang Y., Li T., Kim J., Chim C.-Y., Galli G., Wang F. (2010). Emerging Photoluminescence in Monolayer MoS_2_. Nano Lett..

[157] Novoselov K.S., Jiang D., Schedin F., Booth T.J., Khotkevich V.V., Morozov S.V., Geim A.K. (2005). Two-dimensional atomic crystals. Proc. Natl. Acad. Sci. USA.

[158] Radisavljevic B., Radenovic A., Brivio J., Giacometti V., Kis A. (2011). Single-layer MoS_2_ transistor. Nat. Nanotechnol..

[159] Zhao S., Xue J., Kang W. (2014). Gas adsorption on MoS_2_ monolayer from first-principles calculations. Chem. Phys. Lett..

[160] Shokri A., Salami N. (2016). Gas sensor based on MoS_2_ monolayer. Sens. Actuators B-Chem..

[161] Cho B., Kim A.R., Park Y., Yoon J., Lee Y.-J., Lee S., Yoo T.J., Kang C.G., Lee B.H., Ko H.C. (2015). Bifunctional Sensing Characteristic of Chemical Vapor Deposition Synthesized Atomic-Layered MoS_2_. ACS Appl. Mater. Interfaces.

[162] Liu B., Chen L., Liu G., Abbas A.N., Fathi M., Zhou C. (2014). High-Performance Chemical Sensing Usng Shottky-Contacted Chemical Vapor Deposition Grown Monolayer MoS_2_ Transistors. ACS Nano.

[163] Li H., Yin Z., He Q., Li H., Huang X., Lu G., Fam D.W.H., Tok A.I.Y., Zhang Q., Zhang H. (2012). Fabrication of Single- and Multilayer MoS_2_ Film-Based Field-Effect Transistors for Sensing NO at Room Temperature. Small.

[164] Donarelli M., Prezioso S., Perrozzi F., Bisti F., Nardone M., Giancaterini L., Cantalini C., Ottaviano L. (2015). Response to NO_2_ and other gases of resistive chemically exfoliated MoS_2_-based gas sensors. Sens. Actuators B-Chem..

[165] Zhang S.-L., Choi H.-H., Yue H.-Y., Yang W.-C. (2014). Controlled exfoliation of molybdenum disulfide for developing thin film humidity sensor. Curr. Appl. Phys..

[166] Late D.J., Huang Y.-K., Liu B., Acharya J., Shirodkar S.N., Luo J., Yan A., Charles D., Waghmare U.V., Dravid V.P. (2013). Sensing Behavior of Atomically Thin-Layered MoS_2_ Transistors. ACS Nano.

[167] Perkins F.K., Friedman A.L., Cobas E., Campbell P.M., Jernigan G.G., Jonker B.T. (2013). Chemical Vapor Sensing with Monolayer MoS_2_. Nano Lett..

[168] He Q., Zeng Z., Yin Z., Li H., Wu S., Huang X., Zhang H. (2012). Fabrication of Flexible MoS_2_ Thin-Film Transistor Arrays for Practical Gas-Sensing Applications. Small.

[169] Liu Y.J., Hao L.Z., Gao W., Liu Y.M., Li G.X., Xue Q.Z., Guo W.Y., Yu L.Q., Wu Z.P., Liu X.H. (2015). Growth and humidity-dependent electrical properties of bulk-like MoS_2_ thin films on Si. RSC Adv..

[170] Lee K., Gatensby R., McEvoy N., Hallam T., Duesberg G.S. (2013). High-Performance Sensors Based on Molybdenum Disulfide Thin Films. Adv. Mater..

[171] Koh E.W.K., Chiu C.H., Lim Y.K., Zhang Y.-W., Pan H. (2012). Hydrogen adsorption on and diffusion through MoS_2_ monolayer: First-principles studies. Int. J. Hydrogen Energy.

[172] Yu N., Wang L., Li M., Sun X., Hou T., Li Y. (2015). Molibdenum disulfide as a highly efficient adsorbent for non-polar gases. Phys. Chem. Chem. Phys..

[173] Cho S.-Y., Kim S.J., Lee Y., Kim J.-S., Jung W.-B., Yoo H.-W., Kim J., Jung H.-T. (2015). Highly Enhanced Gas Adsorption Properties in Vertically Aligned MoS_2_ Layers. ACS Nano.

[174] Yan H., Song P., Zhang S., Yang Z., Wang Q. (2015). Dispersed SnO_2_ nanoparticles on MoS_2_ nanosheets for superior gas-sensing performances to ethanol. RSC Adv..

[175] Cui S., Wen Z., Huang X., Chang J., Chen J. (2015). Stabilizing MoS_2_ Nanosheets through SnO_2_ Nanocrystal Decoration for High-Performance Gas Sensing in Air. Small.

[176] Yan H., Song P., Zhang S., Yang Z., Wang Q. (2016). Facile synthesis, characterization and gas sensing performance of ZnO nanoparticles-coated MoS_2_ nanosheets. J. Alloys Compd..

[177] Yan H., Song P., Zhang S., Zhang J., Yang Z., Wang Q. (2016). A low temperature gas sensor based on Au-loaded MoS_2_ hierarchical nanostructures for detecting ammonia. Ceram. Int..

[178] Cho S.-Y., Koh H.-J., Yoo H.-W., Kim J.-S., Jung H.-T. (2017). Tunable Volatile-Organic-Compound Sensor by Using Au Nanoparticle Incorporation on MoS_2_. ACS Sens..

[179] Sarkar D., Xie X., Kang J., Zhang H., Liu W., Navarrete J., Moskovits M., Banerjee K. (2015). Functionalization of Transition Metal Dichalcogenides with Metallic Nanoparticles: Implications for Doping and Gas-Sensing. Nano Lett..

[180] Cho B., Yoon J., Lim S.K., Kim A.R., Choi S.-Y., Kim D.-H., Lee K.H., Lee B.H., Ko H.C., Hahm M.G. (2015). Metal Decoration Effects on the Gas-Sensing Properties of 2D Hybrid-Structures on Flexible Substrates. Sensors.

[181] Baek D.-H., Kim J. (2017). MoS_2_ gas sensor functionalized by Pd for the detection of hydrogen. Sens. Actuators B-Chem..

[182] Kuru C., Choi C., Kargar A., Choi D., Kim Y.J., Liu C.H., Yavuz S., Jin S. (2015). MoS_2_ Nanosheet-Pd Nanoparticle Composite for Highly Sensitive Room Temperature Detection of Hydrogen. Adv. Sci..

[183] Cho B., Yoon J., Lim S.K., Kim A.R., Kim D.-H., Park S.-G., Kwon J.-D., Lee Y.-J., Lee K.-H., Lee B.H. (2015). Chemical Sensing of 2D Graphene/MoS_2_ Heterostructure device. ACS Appl. Mater. Interfaces.

[184] Zhou Y., Liu G., Zhu X., Guo Y. (2017). Ultrasensitive NO_2_ gas sensing based on rGO/MoS_2_ nanocomposite film at low temperature. Sens. Actuators B-Chem..

[185] Niu Y., Wang R., Jiao W., Ding G., Hao L., Yang F., He X. (2015). MoS_2_ graphene fiber based gas sensing devices. Carbon.

[186] Liu G., Rumyantsev S.L., Jiang C., Shur M.S., Balandin A.A. (2015). Selective Gas Sensing With h-BN Capped MoS_2_ Heterostructure Thin-Film Transistors. IEEE Electron Device Lett..

[187] Nayeri M., Moradinasab M., Fathipour M. (2018). The transport and optical sensing properties of MoS_2_, MoSe_2_, WS_2_ and WSe_2_ semiconducting transition metal dichalcogenides. Semicond. Sci. Technol..

[188] Tongay S., Zhou J., Ataca C., Liu J., Kang J.S., Matthews T.S., You L., Li J., Grossman J.C., Wu J. (2013). Broad-Range Modulation of Light Emission in Two-Dimensional Semiconductors by Molecular Physisorption Gating. Nano Lett..

[189] Bui V.Q., Pam T.-T., Le D.A., Thi C.M., Le M.H. (2015). A first-principles investigation of various gas (CO, H_2_O, NO, and O_2_) absorptions on a WS_2_ monolayer: Stability and electronic properties. J. Phys. Condens. Matter.

[190] Zhou C.J., Yang W.H., Wu Y.P., Lin W., Zhu H.L. (2015). Theoretical study of the interaction of electron donor and acceptor molecules with monolayer WS_2_. J. Phys. D Appl. Phys..

[191] Pawbake A.S., Waykar R.G., Late D.J., Jadkar S.R. (2016). Highly Transparent Wafer-Scale Synthesis of Crystalline WS_2_ Nanoparticle Thin Film for Photodetector and Humidity-Sensing Applications. ACS Appl. Mater. Interfaces.

[192] O’Brien M., Lee K., Morrish R., Berner N.C., McEvoy N., Wolden C.A., Duesberg G.S. (2014). Plasma assisted synthesis of WS_2_ for gas sensing applications. Chem. Phys. Lett..

[193] Perrozzi F., Emamjomeh S.M., Paolucci V., Taglieri G., Ottaviano L., Cantalini C. (2017). Thermal stability of WS_2_ flakes and gas sensing properties of WS_2_/WO_3_ composite to H_2_, NH_3_ and NO_2_. Sens. Actuators B-Chem..

[194] Yan W., Harley-Trochimczyk A., Long H., Chan L., Pham T., Hu M., Qin Y., Zettl A., Carraro C., Worsley M.A. (2017). Conductometric gas sensing behavior of WS_2_ aerogel. FlatChem.

[195] Cha J.-H., Choi S.-J., Yu S., Kim I.-D. (2017). 2D WS_2_-edge functionalized multi-channel carbon nanofibers: Effect of WS_2_ edge-abundant structure on room temperature NO_2_ sensing. J. Mater. Chem. A.

[196] Yan W., Worsley M.A., Pham T., Zettl A., Carraro C., Maboudian R. (2018). Effects of ambient humidity and temperature on the NO_2_ sensing characteristics of WS_2_/graphene aerogel. Appl. Surf. Sci..

[197] Ko K.Y., Song J.-G., Kim Y., Choi T., Shin S., Lee C.W., Lee K., Koo J., Lee H., Kim J. (2016). Improvement of Gas-Sensing Performance of Large Area Tungsten Disulfide Nanosheets by Surface Functionalization. ACS Nano.

[198] Kuru C., Choi D., Kargar A., Liu C.H., Yavuz S., Choi C., Jin S., Bandaru P.R. (2016). High-performance flexible hydrogen sensor made of WS_2_ nanosheet-Pd nanoparticle composite film. Nanotechnology.

[199] Ouyang C., Chen Y., Qin Z., Zeng D., Zhang J., Wang H., Xie C. (2018). Two-dimensional WS_2_-based nanosheets modified by Pt quantum dots for enhanced room temperature NH_3_ sensing properties. Appl. Surf. Sci..

[200] Qin Z., Ouyang C., Zhang J., Wan L., Wang S., Xie C., Zeng D. (2017). 2D WS_2_ nanosheets with TiO_2_ quantum dots decoration for high-performance ammonia gas sensing at room temperature. Sens. Actuators B-Chem..

[201] Huo N., Yang S., Wei Z., Li S.-S., Xia J.-B., Li J. (2014). Photoresponsive and Gas Sensing Field-Effect Transistor based on Multilayer WS_2_ Nanoflakes. Sci. Rep..

[202] Guo Z., Zhang H., Lu S., Wang Z., Tang S., Shao J., Sun Z., Xie H., Wang H., Yu X.-F. (2015). From Black Phosphorus to Phosphorene: Basic Solvent Exfoliation, Evolution of Raman Scattering, and Applications to Ultrafast Photonics. Adv. Funct. Mater..

[203] Castellanos-Gomez A., Vicarelli L., Prada E., Island J.O., Narasimha-Acharya K.L., Blanter S.I., Groenendijk D.J., Buscema M., Steele G.A., Alvarez J.V. (2014). Isolation and characterization of few-layer black phosphorus. 2D Mater..

[204] Abbas A.N., Liu B., Chen L., Ma Y., Cong S., Aroonyadet N., Köpf M., Nilges T., Zhou C. (2015). Black Phosphorus Gas Sensors. ACS Nano.

[205] Woomer A.H., Farnsworth T.W., Hu J., Wells R.A., Donley C.L., Warren S.C. (2015). Phosphorene: Synthesis, Scale-Up, and Quantitative Optical Spectroscopy. ACS Nano.

[206] Cai Y., Ke Q., Zhang G., Zhang Y.-W. (2015). Energetics, Charge Transfer, and Magnetism of Small Molecules Physisorbed on Phosphorene. J. Phys. Chem. C.

[207] Kou L., Frauenheim T., Chen C. (2014). Phosphorene as a Superior Gas Sensor: Selective Adsorption and Distinct I-V Response. J. Phys. Chem. Lett..

[208] Yang A.-J., Wang D.-W., Wang X.-H., Chu J.-F., Lv P.-L., Liu Y., Rong M.-Z. (2017). Phosphorene: A Promising Candidate for Highly Sensitive and Selective SF_6_ Decomposition Gas Sensors. IEEE Electron. Device Lett..

[209] Wood J.D., Wells S.A., Jariwala D., Chen K.-S., Cho E.K., Sangwan V.K., Liu X., Lauhon L.J., Marks T.J., Hersam M.C. (2014). Effective Passivation of Exfoliated Black Phosphorus Transistors against Ambient Degradation. Nano Lett..

[210] Cui S., Pu H., Wells S.A., Wen Z., Mao S., Chang J., Hersam M.C., Chen J. (2015). Ultrahigh sensitivity and layer-dependent sensing performance of phosphorene-based gas sensors. Nat. Commun..

[211] Lee G., Kim S., Jung S., Jang S., Kim J. (2017). Suspended black phosphorus nanosheet gas sensor. Sens. Actuators B-Chem..

[212] Donarelli M., Ottaviano L., Giancaterini L., Fioravanti G., Perrozzi F., Cantalini C. (2016). Exfoliated black phosphorus gas sensing properties at room temperature. 2D Mater..

[213] Cho S.-Y., Lee Y., Koh H.-J., Jung H., Kim J.-S., Yoo H.W., Kim J., Jung H.-T. (2016). Superior Chemical Sensing Performance of Black Phosphorus: Comparison with MoS_2_ and Graphene. Adv. Mater..

[214] Hanlon D., Backes C., Doherty E., Cucinotta C.S., Berner N.C., Boland C., Lee K., Harvey A., Lynch P., Gholamvand Z. (2015). Liquid exfoliation of solvent-stabilized few-layer black phosphorus for applications beyond electronics. Nat. Commun..

[215] Wang L., Sofer Z., Pumera M. (2015). Voltammetry of Layered Black Phosphorus: Electrochemistry of Multilayer Phosphorene. ChemElectroChem.

[216] Mayorga-Martinez C.C., Sofer Z., Pumera M. (2015). Layered Black Phosphorus as a Selective Vapor Sensor. Angew. Chem. Int. Ed..

[217] Erande M.B., Pawar M.S., Late D.J. (2016). Humidity Sensing and Photodetection Behavior of Electrochemically Exfoliated Atomically Thin-Layered Black Phosphorus Nanosheets. ACS Appl. Mater. Interfaces.

[218] Late D.J. (2016). Liquid exfoliation of black phosphorus nanosheets and its application as humidity sensor. Microporous Mesoporous Mater..

[219] Yasaei P., Behranginia A., Foroozan T., Asadi M., Kim K., Khalili-Araghi F., Salehi-Khojin A. (2015). Stable and Selective Humidity Sensing Using Stacked Black Phosphorus Flakes. ACS Nano.

[220] Miao J., Cai L., Zhang S., Nah J., Yeom J., Wang C. (2017). Air-Stable Humidity Sensor Using Few-Layer Black Phosphorus. ACS Appl. Mater. Interfaces.

[221] Yao Y., Zhang H., Sun J., Ma W., Li L., Li W., Du J. (2017). Novel QCM humidity sensors using stacked black phosphorus nanosheets as sensing film. Sens. Actuators B-Chem..

[222] Lei S.Y., Yu Z.Y., Shen H.Y., Sun X.L., Wan N., Yu H. (2018). CO Adsorption on Metal-Decorated Phosphorene. ACS Omega.

[223] Lee G., Jung S., Jang S., Kim J. (2017). Platinum-functionalized black phosphorus hydrogen sensors. Appl. Phys. Lett..

[224] Cho S.-Y., Koh H.-J., Yoo H.-W., Jung H.-T. (2017). Tunable Chemical Sensing Performance of Black Phosphorus by Controlled Functionalization with Noble Metals. Chem. Mater..

